# DNA Metabarcoding as a Tool to Study Plankton Responses to Warming and Salinity Change in Mesocosms

**DOI:** 10.1002/ece3.72125

**Published:** 2025-09-15

**Authors:** Clio Abbie Marjorie Hall, Nicolas Henry, Oriol Canals, Gianina Consing, Naiara Rodríguez‐Ezpeleta, Aleksandra M. Lewandowska

**Affiliations:** ^1^ Tvärminne Zoological Station University of Helsinki Hanko Finland; ^2^ CNRS, Sorbonne Université, FR2424, ABiMS, Station Biologique de Roscoff Roscoff France; ^3^ AZTI, Marine Research Basque Research and Technology Alliance (BRTA) Sukarrieta Bizkaia Spain; ^4^ GEOMAR Helmholtz Centre for Ocean Research Kiel Kiel Germany

## Abstract

Climate change is transforming marine ecosystems, with rising temperatures and changing salinity patterns expected to reshape plankton communities in the Baltic Sea. As key components of marine food webs and biogeochemical cycles, plankton are highly sensitive to environmental change. Here, we examined the effects of warming and salinity change on plankton communities using a mesocosm experiment at the Tvärminne Zoological Station, Finland. We employed both traditional microscopy‐based identification and DNA metabarcoding (18S rRNA and COI markers) to assess shifts in phytoplankton, ciliates and mesozooplankton. Our findings indicate that salinity primarily affected higher trophic levels, while warming influenced lower ones. Warmer conditions increased community evenness and favoured mixotrophic and heterotrophic taxa, whereas salinity effects were most pronounced in rotifers and copepods, reflecting species‐specific tolerances. Interactive effects varied, with salinity sometimes buffering warming impacts and other times intensifying them, highlighting complex stressor interactions. Microscopy allowed for a more precise quantification of plankton abundance, whereas metabarcoding captured a broader taxonomic diversity. Our results suggest that, within the tested salinity range (3–10.5 PSU), higher salinities supported a more classical marine food web structure, characterised by larger and more complex zooplankton such as copepods. In contrast, freshening and warming conditions were associated with shifts towards smaller, mixotrophic and bloom‐forming plankton taxa, with potential consequences for ecosystem functioning. This study highlights metabarcoding's value in mesocosm research while emphasising the need to refine molecular techniques for ecological interpretations.

## Graphical abstract

To assess the impact of future climate change on Baltic Sea plankton communities, we conducted a mesocosm experiment manipulating temperature and salinity, using both microscopy‐based and DNA metabarcoding for plankton identification. Our results show that warming primarily influenced lower trophic levels, increasing community evenness and favouring mixotrophic and heterotrophic taxa, while salinity effects were strongest in rotifers and copepods. While microscopy provided precise abundance estimates, metabarcoding captured a broader taxonomic diversity, revealing nuanced shifts in community composition. These findings highlight the complex nature of climate‐driven changes in plankton communities and underscore the complementary strengths of microscopy and metabarcoding for biodiversity monitoring.

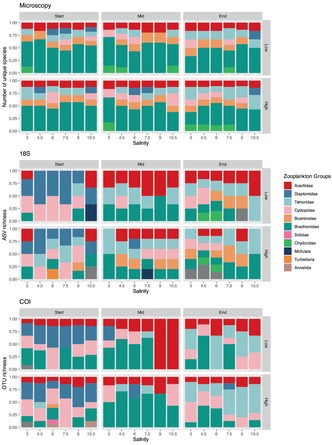

## 1 Introduction

The Earth's climate is changing rapidly, with rising temperatures and altered hydrological cycles affecting marine ecosystems (Travis 2003; Andersson et al. 2015; Ahola et al. 2021). One often overlooked but increasingly significant stressor in both marine and freshwater systems is the alteration of water salinity, driven primarily by climate‐induced changes in precipitation, freshwater runoff and ice melt (Helm et al. 2010; Ross et al. 2021), as well as anthropogenic activities such as land‐use change and road salting (Hintz et al. 2022). Globally, both salinisation and desalinisation trends are being observed across estuaries, coastal zones and semi‐enclosed seas, shaped by hydrological variability and regional environmental conditions (Herbert et al. 2015; Moffett et al. 2023). In the Baltic Sea, salinity is generally projected to decline by approximately 2 PSU and temperature to rise by 2°C to 4°C by the end of the century, largely due to increased precipitation and reduced saltwater inflow (Meier et al. 2006; Vuorinen et al. 2015; Gröger et al. 2019). However, salinity projections remain subject to considerable spatial and temporal uncertainty, with some models predicting region‐specific increases under alternative wind regimes, inflow dynamics or circulation patterns (Meier et al. 2006; Vuorinen et al. 2015). These shifts in temperature and salinity can substantially alter the distribution, phenology, abundance, composition and trophic interactions of plankton (Winder et al. 2012; Larson and Belovsky 2013; Boyd et al. 2014; Rasconi et al. 2017; Gittings et al. 2018; Murphy et al. 2020; Hall and Lewandowska 2022; Park et al. 2023).

Plankton form the base of the marine food web, acting as primary producers and key links between trophic levels. Changes in their communities can have cascading effects on higher trophic levels (Ehrlich and Gaedke 2020; Schmidt et al. 2020), including commercially important fish (Abo‐Taleb 2019), seabirds (Lauria et al. 2013) and marine mammals (Fu et al. 2020). Plankton also play a crucial role in global biogeochemical cycles, particularly through the biological carbon pump, nutrient recycling and oxygen production, thereby influencing Earth's climate (Falkowski et al. 1998) and maintaining the productivity of marine ecosystems (Robinson et al. 2017). Because of their short generation times and high diversity, plankton rapidly reflect environmental perturbations (Marvá et al. 2014; Busseni et al. 2019), making them sensitive indicators of ecosystem health (Li et al. 2022) and useful for studying the impacts of environmental variability and climate change over short and long timescales (Busseni et al. 2019). Individual plankton groups also vary in their susceptibility to environmental stressors, so responses may be non‐linear and amplified throughout the food web; therefore, they will react to even the most subtle environmental changes (Taylor et al. 2002).

Warming can alter plankton metabolic rates, growth and reproductive success (Garcia‐Corral et al. 2017; Vázquez‐Domínguez et al. 2012; Yvon‐Durocher et al. 2015) as well as promote cyanobacterial growth, leading to increased biomass production and toxic algal blooms that disrupt ecosystems (Lürling et al. 2017; Courboulès et al. 2021). Changes in salinity can affect osmoregulation (Tee et al. 2021), nutrient availability and community interactions (Francis et al. 2012). However, the combined effects of warming and salinity remain poorly understood, and stressors may interact in complex ways, producing synergistic or antagonistic effects (Maher et al. 2019). For example, warming can enhance phytoplankton growth (Hays et al. 2005), but salinity intolerance may exacerbate physiological stress, reducing growth rates (Hernando et al. 2015). Similarly, the combined effects of warming and reduced salinity could affect the osmoregulation abilities of zooplankton, affecting reproductive success and survival (Roddie et al. 1984). Such changes may reshape plankton community structure, favouring certain species, altering species dominance and community composition (Benedetti et al. 2021; Hall and Lewandowska 2022). This can trigger cascading effects that impact overall ecosystem functioning (Viitasalo and Bonsdorff 2022).

Despite their value in assessing ecosystem responses to environmental change, studying plankton communities presents challenges, particularly in taxonomic identification. Traditional microscopy‐based methods are difficult due to the high diversity and complexity of plankton, requiring expert knowledge across groups ranging from bacteria and phytoplankton to zooplankton. Identification is often time‐consuming, labour‐intensive and sometimes even impossible to distinguish species at juvenile stages, such as copepods and their nauplii. In addition, pico‐ and nanoplankton often lack distinguishing morphological features, meaning important primary producers and nano‐consumers may be overlooked in analysis (Ishizaka et al. 1997). These challenges are further amplified in large‐scale or long‐term studies requiring the analysis of vast quantities of samples. Recent advancements in DNA metabarcoding offer a solution to many challenges in morphology‐based identification (Abad et al. 2016; Santoferrara 2019; Novotny et al. 2023). This high‐throughput method enables efficient biodiversity assessment across trophic levels in complex samples (Fahner et al. 2016; Rivera et al. 2020; Prié et al. 2023) by amplifying and sequencing genetic markers like 18S rRNA and COI. With increasing sequencing capacity and declining costs, metabarcoding is ideal for diversity monitoring (Gran‐Stadniczeñko et al. 2019). The 18S rRNA marker is widely used in eukaryotic and multi‐trophic research (Zamora‐Terol et al. 2020). As a highly conserved gene across eukaryotes, 18S is particularly suitable for protist studies but also effective for animals, making it valuable for plankton research (MacNeil et al. 2022). However, 18S—particularly the V9 region—often clusters multiple species into a single ASV, especially in animals (Clarke et al. 2017). In contrast, the COI marker can provide higher taxonomic resolution for animals, making it particularly useful for identifying higher trophic‐level eukaryotes like zooplankton (Suter et al. 2021). Since COI databases are well‐developed for animals, while 18S provides broader protist coverage, combining both markers offers a more comprehensive view of plankton diversity and food web dynamics (Stefanni et al. 2018; Zhang et al. 2018).

The use of metabarcoding has significant implications for plankton research, enabling rapid assessments of community composition, species shifts, invasive species tracking and environmental stressors like climate change and pollution (Suter et al. 2021). It has already been instrumental in elucidating complex trophic interactions (Pompanon et al. 2012; Roslin and Majaneva 2016), including within plankton food webs in the Baltic Sea (Novotny et al. 2021). However, like any emerging technology, rigorous validation is essential to avoid biases in sampling, sequencing and data interpretation, which can lead to inaccurate ecological interpretations (Bálint et al. 2018; Dickie et al. 2018). To date, metabarcoding has primarily been used for species identification in natural environments. However, its advantages can undoubtedly be extended to experimental conditions (e.g., mesocosms), for which few studies exist (Ray et al. 2016; Bourque et al. 2023; Min et al. 2023). By integrating metabarcoding into controlled experiments, researchers can better address uncertainties related to methodological biases through sample replication and comparison. This is particularly important because, in natural settings, DNA signals can be distorted by hydrodynamics and temporal biases. These issues can be effectively controlled within mesocosms, providing a more reliable context for analysis.

To explore these dynamics, we conducted an experiment investigating the effects of salinity changes and warming on plankton communities in the Baltic Sea, while also assessing the potential of metabarcoding in mesocosm experiments. We utilised an indoor mesocosm facility—a well‐established method for studying plankton community responses to environmental change due to its capacity for manipulation and replication (Woodward et al. 2012; Hall and Lewandowska 2022). The Baltic Sea serves as an ideal research location, as its brackish waters foster interactions between freshwater and marine plankton communities (Ojaveer et al. 2010) with composition strongly shaped by seasonal and biogeographic dynamics (Serandour et al. 2024). These complexities underscore the value of controlled mesocosm experiments in disentangling direct environmental effects from broader ecological variation. The objectives of this study were to examine how plankton communities respond to climate‐induced changes in salinity and temperature and to assess the capacity of metabarcoding to detect these ecological responses. Specifically, the study addressed the following research questions:

1. How do changes in temperature and salinity, and their interaction, affect plankton communities with a focus on phytoplankton, ciliates and mesozooplankton groups? *We hypothesised that temperature and salinity would have distinct effects on different plankton groups, with warming favouring smaller, faster‐growing taxa and salinity filtering plankton based on tolerance. We expected interactive effects to either amplify or buffer these responses*.
2. To what extent do DNA metabarcoding and microscopy provide complementary or divergent perspectives on community composition? *We hypothesised that DNA metabarcoding using 18S and COI markers would detect greater taxonomic diversity than morphology‐based identification, particularly for small or cryptic taxa, but would be less sensitive to treatment effects due to limited quantitative resolution*.

## 2 Methods

We conducted an indoor mesocosm experiment in September–October 2022 at Tvärminne Zoological Station, Finland (59.84° N, 23.25° E), to investigate the effects of warming and salinity changes on marine plankton food webs. The setup included 12 manually mixed mesocosms (600 L; 0.5 m width, 1.25 m length, 1 m depth; 200 μm LDPE troughs). A continuous salinity gradient (3 [control], 4.5, 6, 7.5, 9, 10.5 PSU) was applied at two temperature levels (17°C—ambient; 21°C—projected warming), based on Baltic Sea climate projections (Meier et al. 2012). Salinity was implemented as a continuous gradient rather than discrete treatments, allowing detection of ecological responses across a realistic environmental range. This design reflects natural salinity transitions in the Baltic Sea and supports valid statistical inference using continuous predictors and random effects in mixed models (Norberg et al. 2001; Benedetti‐Cecchi 2001). The selected salinity range covers surface conditions observed across much of the Baltic Sea, from the lower‐salinity Gulf of Finland and Bothnian Bay to the more saline central and western basins (European Union‐Copernicus Marine Service 2018). Target treatment values were designed relative to ambient salinity (~5.5 PSU), using real‐time data from the Ångbåtsbryggan MONICOAST monitoring buoy, ensuring local relevance during the experimental period.

To create ecologically representative plankton communities, each mesocosm was filled with 300 L freshwater from Gennarbyviken Reservoir (59.92° N, 23.21° E; 0 PSU), a waterbody hydrologically isolated from the sea since 1957 but still supporting a diverse plankton community of marine origin and 300 L brackish water (5.5 PSU) from Tvärminne Bay. The combined source waters provided a natural assemblage of coexisting plankton communities spanning a salinity gradient, reflecting typical late‐summer diversity in the region. Plankton were collected via sub‐surface hoses and transported in a water truck and transferred to mesocosms using a low‐pressure gravity flow with no mechanical pumping to minimise physical stress. Microscopy checks confirmed that plankton survived transportation and that communities were comparable across mesocosms post‐filling.

On DOY 249 to 250, Instant Ocean salt was added in batches to non‐control mesocosms. Salt was pre‐dissolved in 10 L of extracted water and gently reintroduced into each mesocosm using a Secchi disk to facilitate even mixing. The control mesocosm was agitated similarly. Salinity was verified the next day to ensure target levels were achieved. Natural diel light cycles were simulated using one full‐spectrum LED lamp per mesocosm (200 W, AquaMedic, controlled by ProfiLux 4, GHL), suspended 1 m above the water surface. The light regime was programmed to replicate the natural daylight cycle (sunrise, solar noon and sunset) based on in situ light conditions recorded on the first day of the experiment (DOY 249), with a solar noon intensity of 150 μmol photons m^−2^ s^−1^. The experiment ran for 30 days and was terminated on DOY 279.

### 2.1 Sample Collection

We measured all abiotic and biotic parameters at 1 m depth from the middle of the mesocosm. Temperature (°C), salinity, fluorescence (relative fluorescence units, RFU) and dissolved oxygen (mg O_2_/L) were measured every 2 days using a portable calibrated digital water metre (MU 6100 H, VWR). Samples for bacteria, nanoflagellates and phytoplankton abundance, as well as nutrient concentrations, were taken every 2 days. Four litre of water from each mesocosm was taken every 2 days using a water sampler and placed into a 10 L plastic container for transportation to the laboratory. The canister was stored in a cold room before gentle mixing was applied, and samples for different uses were syphoned off from the container (chlorophyll‐a, dissolved nutrients, bacteria, etc.). For mesozooplankton, samples were taken every 6 days; a 50 μm plankton net was dragged from 1 m depth from the centre of each mesocosm and then flushed into 250 mL plastic bottles with 50% ethanol for transportation to the laboratory. For microzooplankton, samples were syphoned off from the common plastic container. For a direct comparison with metabarcoding, only samples for phytoplankton, ciliates and zooplankton taken every 2 weeks were analysed. DNA samples were taken every 2 weeks from all mesocosms following slightly modified methods from chapters 3 and 4.2 from Minamoto et al. (2021). We filtered 1000 mL of water onto a 47 mm diameter, 0.7 μm pore size GF/F (Whatman, USA) using a vacuum pump. All filters were transferred to 1.5 mL Eppendorf tubes (DNA LoBind) and stored at −80°C before being transported on dry ice to the Molecular Ecology and Systematics Laboratory, Finland, for sequencing.

### 2.2 Morphology‐Based Plankton Identification

#### 2.2.1 Phytoplankton

Phytoplankton samples of 100 mL were taken from the main sample canister and preserved with Lugol's iodine (1%). Phytoplankton were counted using 50 mL Utermöhl chambers (Utermöhl 1958). Settling time differs depending on chamber volume: 10 mL to 8 h; 25 mL to 18 h; 50 mL to 24 h and identified to genus level using an inverted microscope (Olympus CKx41). The enumeration method was conducted with at least 20 fields at (20× and 40× objective) and two transects (10× objective). If the same species were particularly abundant, up to 500 cells were counted and then counting was stopped for that species. Final abundance is given in cells per millilitre.

#### 2.2.2 Ciliates

Ciliate samples of 100 mL were taken from the main sample canister and stained with Lugol's iodine (1%) to preserve them for further analysis. Ciliates were counted using 50 mL Utermöhl chambers after a settling time of 24 to 48 h (Freibott et al. 2014) and identified to genus level using an inverted microscope (Olympus CKx41) with the 40× objective. The entire chamber was counted. Final abundance is given in individuals per litre.

#### 2.2.3 Zooplankton

To quantify zooplankton abundance, the sample was settled in 50 mL Utermöhl chambers for 30 min and counted using an Olympus CK30 at ×10 and ×40 magnification using an inverted microscope technique (Lund et al. 1958). Depending on the density of the sample, a Folsom plankton splitter was used to split the sample into ¼, ½, or ¾ of the original sample. Identification to genus level (and species where possible) was made using Telesh et al. (2008). Few adult copepods were recorded; therefore, we grouped adults and copepodites together in analyses. Copepod nauplii were analysed separately. Final abundance is given in individuals per litre.

### 2.3 Metabarcoding‐Based Plankton Identification

#### 2.3.1 DNA Extraction and Amplicon Library Preparation

DNA from filters was extracted using DNeasy Blood and Tissue kits (Qiagen, Germantown, MD) following the protocol from Minamoto et al. (2021) with some modifications that included using Buffer ATL instead of AL and increasing the incubation time to 6 h. DNA extraction concentrations were quantified using a NanoDrop 1000 spectrophotometer (ThermoFisher Scientific, Waltham, MA). The integrity of extracted genomic DNA was assessed by electrophoresis in 0.7% agarose.

We amplified the cytochrome c oxidase subunit I (COI) gene using the primers mlCOIintF and HCO2198 (Leray et al. 2013; Folmer et al. 1994) and the V9 region of the 18S rRNA gene (18S) using the primers 1391F and EukBr (Amaral‐Zettler et al. 2009; Medlin et al. 1988) (Table 1). One 25 μL PCR reaction consisted of 1× Phusion Green HF buffer (Thermo Fisher Scientific, Waltham, MA, USA), 0.2 mM dNTP mix (Thermo Fisher Scientific), 0.5 μM of each primer, 0.5 U of Phusion High‐Fidelity II DNA polymerase (Thermo Fisher Scientific), 2.5% DMSO and approximately 30–90 ng of DNA. The thermal profile was 98°C for 30 s followed by 20 cycles of denaturation at 98°C for 10 s, annealing at 60°C for 30 s and extension at 72°C for 10 s, before ending with 72°C for 5 min and 4°C for 5 min. Products from the first PCR were treated with Exonuclease I and Shrimp Alkaline Phosphatase for 30 min at 37°C to remove excess free primers. 12 μL of the PCR product was used for dual indexing PCR with Phusion Hot‐Start II DNA Polymerase (Thermo Fisher Scientific) using indexing primers (BIDGEN Lab's in‐house design) that have unique combinations of 8 bp i5 and i7 indexes. The thermal profile was 98°C for 30 s followed by 18 cycles of denaturation at 98°C for 10 s, annealing at 65°C for 30 s and extension at 72°C for 10 s, before ending with 72°C for 5 min and 4°C for 5 min. PCR products were finally pooled and purified with MagSi‐NGS plus beads (Magtivio) at a ratio of 0.9×. The library pool was sequenced on the MiSeq (Illumina) with paired‐end reads (326 bp Read 1 and 278 bp Read 2) at a final library concentration of 9.5 pM using a MiSeq V3 600 cycle flow cell (Illumina). Library preparation and sequencing were conducted at the DNA Sequencing and Genomics Laboratory, Institute of Biotechnology, University of Helsinki, Finland.

**TABLE 1:** List of primers used in the study, including their target markers, primer names, sequences and references.

Marker	Primer name	Sequence	References
18S rRNA forward	1391F	GTACACACCGCCCGTC	Amaral‐Zettler et al. (2009)
18S rRNA reverse	EukBr	TGATCCTTCTGCAGGTTCACCTAC	Medlin et al. (1988)
COI forward	mlCOIintF	GGWACWGGWTGAACWGTWTAYCCYCC	Leray et al. (2013)
COI reverse	HCO2198	AAACTTCAGGGTGACCAAAAAATCA	Folmer et al. (1994)

#### 2.3.2 Sequence Quality Assessment and Bioinformatics

Paired‐end reads were trimmed to remove PCR primer sequences using Cutadapt v 3.1 (Martin 2011). Paired‐end reads without both primers were filtered out using the option‐discard‐untrimmed. Plots summarising forward and reverse reads quality were generated for each sample using the function plotQualityProfile() from the R package dada2 (Callahan et al. 2016). Forward and reverse reads were trimmed at position 210 to improve their overall quality. Reads with ambiguous nucleotides or with a maximum number of expected errors (maxEE) superior to 2 were filtered out using the function filterAndTrim() from the R package dada2. For each run, error rates were defined using the function learnErrors(). Reads were denoised using the dada() function. After denoising, forward and reverse reads were merged using mergePairs(). Remaining chimaeras were removed using the function removeBimeraDenovo(). For COI, because of the high level of intragenomic variability, ASVs were clustered in OTUs using Swarm with a local similarity threshold (d) of 4 differences. For both markers, genetically close co‐occurring OTU/ASVs were clustered together using lulu (Frøslev et al. 2017) to remove remaining artefactual OTU/ASVs. Finally, 18S V9 ASVs were taxonomically assigned to PR2 5.0.0 using IDTAXA (40% confidence threshold) for the COI OTUs were assigned to MIDORI2 (GB254) using VSEARCH's command usearch_global (consensus taxonomy of references with a similarity score superior to 80% and comprised between the best hit score and [best hit score] * 99%).

### 2.4 Statistical Analysis

We applied a non‐metric multidimensional scaling (NMDS; metaMDS function, vegan package; Oksanen et al. 2001) analysis based on Bray‐Curtis dissimilarities (vegdist function, vegan package) of Hellinger‐transformed (decostand function, vegan package) read abundance data and microscopy count data to visualise compositional differences between samples at the community level in relation to the response of plankton to salinity and warming over time. Permutational multivariate analysis of variance (PERMANOVA) was used to determine the significance of the treatments and their interactions in explaining the variation in community composition.

To assess changes occurring at the taxon level for microscopy, we conducted linear mixed effects models to test the effect of salinity change and temperature on phytoplankton, ciliate and zooplankton community structure and alpha diversity (species richness, Shannon index, Simpson index and species evenness). Count data for microscopy was log transformed to fulfil normality and variance homogeneity assumptions. Abundance for molecular data was defined as the number of reads per OTU/ASV. Metabarcoding data was rarefied before alpha‐diversity calculations using the rrarefy function (vegan package) to standardise sequencing depth across samples. 18S and COI data were rarefied to a minimum read count of 24,932 and 52,228 reads per sample, respectively. A linear mixed effects model was fitted for microscopy data using restricted maximum likelihood estimation (REML) using package nlme (Pinheiro 2011). The response variable was *x* and the fixed effects were salinity, temperature, their interaction and date. Mesocosm ID was included as a random effect. Model selection was made by using Akaike information criteria (AIC) and autocovariance estimates (ACF). Significance levels were set at *p* < 0.05. Model residuals were checked for homogeneity of variances and normality.

To determine changes at the taxon level for the metabarcoding data, we tested the effects using Linear Models for Differential Abundance Analysis of Microbiome Compositional Data (LinDA: Zhou et al. 2022). LinDA accounts for the compositional nature of sequencing data and reports effects as log fold change (LFC). We applied a prevalence filter of 10% to remove low‐abundance taxa using the Benjamini‐Hochberg (BH) method to correct for multiple testing. Zero inflation was addressed by adding a pseudo‐count of 0.5. The structure of the models was the same as microscopy models. All analyses were performed in R v4.4.2 (R Core Team 2021).

## 3 Results

**FIGURE 1:**
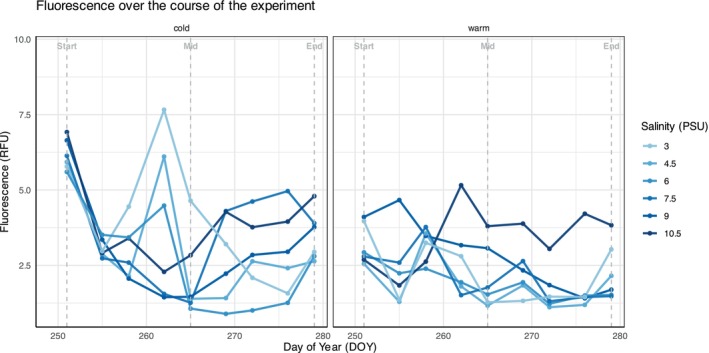
Fluorescence measured as relative fluorescence units (RFU) over the course of the experiment.

Fluorescence levels were higher at lower temperatures during the experiment and showed significant variation over time (Figure 1, Table 2).

**TABLE 2:** Linear model results for fluorescence and plankton group abundances with warming, salinity and time.

Method	Response	Salinity	Temperature	Time	Temp × sal
	Fluorescence	*F* = 2.4573, *p* = 0.1514	*F* = 9.1769, *p* = 0.0143*	*F* = 14.5095, *p* = 0.0002**	
Microscopy	Zooplankton	*F* = 3.74027, *p* = 0.0851	*F* = 0.34267, *p* = 0.5727	*F* = 10.68252, *p* < 0.0001***	
Microscopy	Ciliates	*F* = 1.0301, *p* = 0.3367	*F* = 2.3765, *p* = 0.1576	*F* = 2.6036, *p* = 0.0750	
Microscopy	Phytoplankton	*F* = 6.2388, *p* = 0.0340*	*F* = 4.1568, *p* = 0.0719	*F* = 23.5712, *p* < 0.0001***	
Microscopy	Rotifers	*F* = 6.67374, *p* = 0.0295*	*F* = 0.02324, *p* = 0.8822	*F* = 5.51117, *p* = 0.0044**	
Microscopy	Copepods	*F* = 6.28669, *p* = 0.0335*	*F* = 0.74725, *p* = 0.4098	*F* = 9.19654, P < 0.0001***	
Microscopy	Copepod nauplii	*F* = 8.71908, *p* = 0.0161*	*F* = 0.02243, *p* = 0.8842	*F* = 11.03728, *p* = 0.0005***	
Microscopy	Cladocerans	*F* = 0.088458, *p* = 0.7729	*F* = 0.010548, *p* = 0.9204	*F* = 6.557446, *p* = 0.0019**	
Microscopy	Cyanobacteria	*F* = 12.1121, *p* = 0.0069**	*F* = 0.1461, *p* = 0.7112	*F* = 4.4885, *p* = 0.0115*	
Microscopy	Chlorophyta	*F* = 5.6823, *p* = 0.0410*	*F* = 7.0524, *p* = 0.0262	*F* = 14.9541, *p* < 0.0001***	
Microscopy	Diatoms	*F* = 0.07483, *p* = 0.7906	*F* = 0.54931, *p* = 0.4775	*F* = 5.48338, *p* = 0.0043**	
Microscopy	Dinoflagellates	*F* = 0.01695, *p* = 0.8993	*F* = 2.38926, *p* = 0.1566	*F* = 0.43240, *p* = 0.6493	
Microscopy	Cryptophyta	*F* = 1.82316, *p* = 0.2099	*F* = 16.88443, *p* = 0.0026**	*F* = 7.30670, *p* = 0.0008***	
Microscopy	Ochrophyta	*F* = 0.14021, *p* = 0.7167	*F* = 0.07670, *p* = 0.7881	*F* = 0.27882, *p* = 0.7569	
Microscopy	Haptophyta	*F* = 1.6246, *p* = 0.2344	*F* = 5.2912, *p* = 0.0470*	*F* = 0.0182, *p* = 0.9820	
Microscopy	*Acartia*	*F* = 4.859809, *p* = 0.0586	*F* = 3.768918, *p* = 0.0882	*F* = 8.750538, *p* = 0.0016**	*F* = 6.046234, *p* = 0.0394*
Microscopy	*Prymnesiales*	*F* = 1.6246, *p* = 0.2344	*F* = 5.2912, *p* = 0.0470*	*F* = 0.0182, *p* = 0.9820	
Microscopy	*Strobilidium*	*F* = 2.9622, *p* = 0.1235	*F* = 0.1212, *p* = 0.7367	*F* = 2.7944, *p* = 0.0829	*F* = 7.0244, *p* = 0.0292*
Microscopy	*Balanus*	*F* = 5.117441, *p* = 0.0500	*F* = 0.111933, *p* = 0.7456	*F* = 5.534529, *p* = 0.0113*	
Microscopy	*Brachionus*	*F* = 0.165034, *p* = 0.6941	*F* = 5.864397, *p* = 0.0385*	*F* = 5.847715, *p* = 0.0092**	
Microscopy	*Keratella quadrata*	*F* = 10.19245, *p* = 0.0110*	*F* = 0.02845, *p* = 0.8698	*F* = 13.84230, *p* = 0.0001***	
Microscopy	*Mesocyclops*	*F* = 5.422552, *p* = 0.0448*	*F* = 1.912549, *p* = 0.2000	*F* = 2.957875, *p* = 0.0728	
Microscopy	*Cyclotella choctawhatcheeana*	*F* = 0.1599, *p* = 0.6986	*F* = 6.2824, *p* = 0.0335*	*F* = 3.9128, *p* = 0.0352*	
Microscopy	*Peridiniales*	*F* = 1.79296, *p* = 0.2134	*F* = 9.94372, *p* = 0.0117*	*F* = 4.42267, *p* = 0.0243*	
Microscopy	*Pyramimonas*	*F* = 1.80869, *p* = 0.2116	*F* = 6.27174, *p* = 0.0336*	*F* = 6.04373, *p* = 0.0081**	
Microscopy	*Teleaulax*	*F* = 0.37614, *p* = 0.5549	*F* = 5.70469, *p* = 0.0407*	*F* = 7.67636, *p* = 0.0030**	
Microscopy	*Telonema subtile*	*F* = 0.16588, *p* = 0.6933	*F* = 7.55220, *p* = 0.0225*	*F* = 1.44423, *p* = 0.2574	
Microscopy	*Laboea*	*F* = 0.17636, *p* = 0.6844	*F* = 6.31846, *p* = 0.0331*	*F* = 1.81284, *p* = 0.1867	
Microscopy	*Strombidium*	*F* = 0.00153, *p* = 0.9697	*F* = 6.49938, *p* = 0.0312*	*F* = 3.89737, *p* = 0.0356*	
Microscopy	*Vorticella*	*F* = 6.18527, *p* = 0.0346*	*F* = 0.15539, *p* = 0.7026	*F* = 10.44746, *p* = 0.0006***	
Microscopy	*Monoraphidium contortum*	*F* = 2.3299, *p* = 0.1613	*F* = 12.0949, *p* = 0.0070**	*F* = 0.3450, *p* = 0.7120	
Microscopy	*Pyramimonas*	*F* = 1.80869, *p* = 0.2116	*F* = 6.27174, *p* = 0.0336*	*F* = 6.04373, *p* = 0.0081**	
Microscopy	Flagellates (2‐5um)	*F* = 1.496, *p* = 0.2523	*F* = 6.705, *p* = 0.0292*	*F* = 2.861, *p* = 0.0787	

*Note:* Signficance levels are indicated as *p < 0.05, **p < 0.01 and ***p < 0.001. Only significant temperature x salinity interaction effects are reported for clarity.

### 3.1 Community Level

#### 3.1.1 Beta Diversity

**FIGURE 2:**
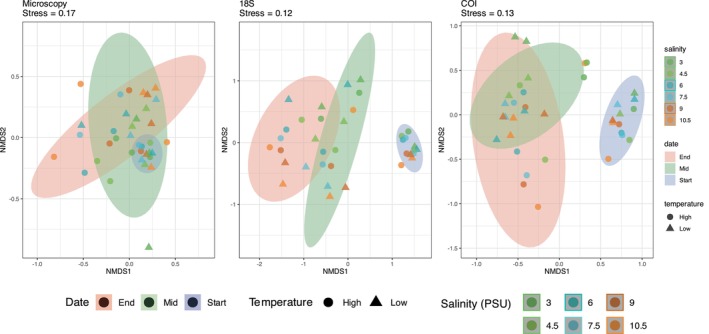
Non‐metric multidimensional scaling (NMDS) plots showing temporal changes in community composition across microscopy, 18S and COI datasets.

NMDS ordination revealed distinct patterns in plankton community distribution over time across 18S, COI and microscopy datasets (Figure 2). The calculated NMDS stress values (0.12 for 18S, 0.13 for COI and 0.17 for microscopy) indicated an acceptable model fit for ecological studies (Clarke 1993). The NMDS plots for 18S and COI metabarcoding show a shift in plankton community composition over time, with a significant temporal transition from the start of the experiment (Table 3). However, neither dataset showed a strong response to salinity or warming. In contrast, microscopy data revealed significant community changes over time as well as a response to warming and salinity (Table 3).

**TABLE 3:** PERMANOVA results from the experiment. *p* values were obtained using 999 permutations of residuals under a reduced model.

Method	Factor	DF	Sum of squares	*R* ^2^	*F*	P(>*F*)
Microscopy	Salinity	1	0.2142	0.04775	2.1673	0.017*
	Temperature	1	0.3498	0.07819	3.5486	0.001**
	Date	2	0.8579	0.19104	4.3353	0.001**
	Residual	31	3.0561	0.68302		
18S	Salinity	1	0.2085	0.0193	1.1469	0.263
	Temperature	1	0.1245	0.01152	0.6847	0.693
	Date	2	4.8356	0.44757	13.2996	0.001**
	Residual	31	5.6357	0.52161		
COI	Salinity	1	0.195	0.02545	1.4828	0.176
	Temperature	1	0.0807	0.01053	0.6133	0.744
	Date	2	3.3105	0.43198	12.5847	0.001**
	Residual	31	4.0774	0.53205		

*Note:* Significance levels are indicated as *p < 0.05, **p < 0.01 and ***p < 0.001.

#### 3.1.2 Alpha Diversity Measures

**FIGURE 3:**
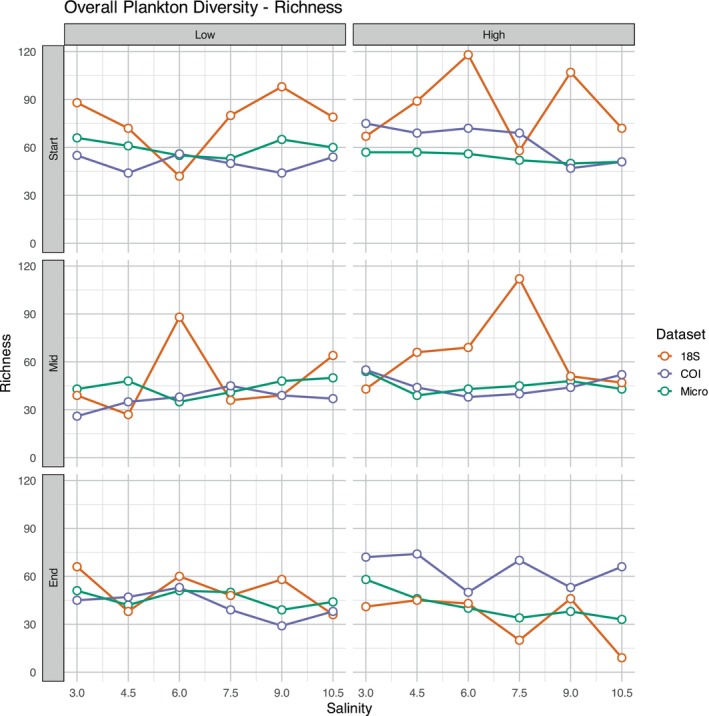
Overall plankton community richness across microscopy (micro), 18S and COI datasets over time (Start, Mid, End), salinity gradient and temperature (Low, High).

Overall, metabarcoding detected higher total ASV/OTU richness than microscopy, with 18S (total ASV richness: 427), COI (total OTU richness: 369) and microscopy (total taxa richness: 146). However, average richness per sample was similar across methods, with 18S averaging 60 ASVs, COI 50 OTUs and microscopy 49 taxa (Figure S8). Diversity patterns differed across datasets, with variations in richness and evenness observed between microscopy and metabarcoding approaches (Table 4, Figure 3, Figures S4–S6). For the microscopy data, community evenness (*all taxa*, Hill 1973) and diversity (Shannon) was influenced by temperature, with a more even and diverse community observed at warmer temperatures (Table 4). The zooplankton community was affected by temperature, having a richer community in higher temperatures (Table 4). The phytoplankton community was affected by salinity, with a richer and more diverse (Shannon) community in low salinities. For COI metabarcoding, total OTU richness was higher in warmer temperatures (Figure 3, Table 4).

**TABLE 4:** Diversity measures with warming, salinity and over time for each of the methods metabarcoding and microscopy.

Method	Response	Salinity	Temperature	Time
Microscopy	Overall community evenness	*F* = 0.0114, *p* = 0.9174	*F* = 6.6755, *p* = 0.0295*	*F* = 1.6087, *p* = 0.2228
Microscopy	Overall community richness	*F* = 4.8076, *p* = 0.0560	*F* = 2.7501, *p* = 0.1316	*F* = 18.1995, *p* < 0.0001***
Microscopy	Overall Shannon diversity	*F* = 0.0144, *p* = 0.9070	*F* = 7.8740, *p* = 0.0205*	*F* = 2.3499, *p* = 0.1189
Microscopy	Overall Simpson diversity	*F* = 0.5053, *p* = 0.4952	*F* = 3.1479, *p* = 0.1098	*F* = 2.7289, *p* = 0.0874
18S	Overall community richness	*F* = 0.04009, *p* = 0.8457	*F* = 0.09400, *p* = 0.7661	*F* = 9.98399, *p* = 0.0008***
18S	Overall community evenness	*F* = 2.0025, *p* = 0.1907	*F* = 0.3407, *p* = 0.5737	*F* = 16.2109, *p* < 0.0001***
18S	Overall Shannon diversity	*F* = 1.6084, *p* = 0.2365	*F* = 0.0448, *p* = 0.8370	*F* = 24.8708, *p* < 0.0001***
18S	Overall Simpson diversity	*F* = 1.9121, *p* = 0.2001	*F* = 1.9019, *p* = 0.2012	*F* = 14.3207, *p* = 0.0001***
COI	Overall community richness	*F* = 3.0115, *p* = 0.1167	*F* = 26.1512, *p* = 0.0006***	*F* = 11.9683, *p* = 0.0003***
COI	Overall community evenness	*F* = 0.3634, *p* = 0.5615	*F* = 1.9209, *p* = 0.1991	*F* = 4.4462, *p* = 0.0239*
COI	Overall Shannon diversity	*F* = 0.7256, *p* = 0.4164	*F* = 0.0137, *p* = 0.9094	*F* = 7.2679, *p* = 0.0038**
COI	Overall Simpson diversity	*F* = 0.0499, *p* = 0.8283	*F* = 1.6433, *p* = 0.2319	*F* = 3.7032, *p* = 0.0411*
18S	Zooplankton richness	*F* = 0.1153, *p* = 0.7420	*F* = 1.8305, *p* = 0.2091	*F* = 0.7366, *p* = 0.4902
18S	Phytoplankton richness	*F* = 1.6184, *p* = 0.2352	*F* = 3.5558, *p* = 0.0920	*F* = 9.4762, *p* = 0.0011**
18S	Ciliate richness	*F* = 0.1867, *p* = 0.6758	*F* = 1.0073, *p* = 0.3418	*F* = 8.2916, *p* = 0.0021**
COI	Zooplankton richness	*F* = 0.0012, *p* = 0.9731	*F* = 0.5616, *p* = 0.4728	*F* = 9.6012, *p* = 0.0010**
COI	Phytoplankton richness	*F* = 0.8541, *p* = 0.3795	*F* = 0.0000, *p* = 1.0000	*F* = 19.9488, *p* < 0.0001***
Microscopy	Zooplankton richness	*F* = 0.0045, *p* = 0.9481	*F* = 5.2352, *p* = 0.0479*	*F* = 9.8933, *p* = 0.0009***
Microscopy	Phytoplankton richness	*F* = 7.1522, *p* = 0.0254*	*F* = 2.7303, *p* = 0.1329	*F* = 19.4451, *p* < 0.0001***
Microscopy	Ciliate richness	*F* = 0.1973, *p* = 0.6674	*F* = 3.1716, *p* = 0.1086	*F* = 1.6771, *p* = 0.2099
18S	Phytoplankton Shannon diversity	*F* = 0.0213, *p* = 0.8871	*F* = 0.3110, *p* = 0.5907	*F* = 1.0066, *p* = 0.3817
18S	Ciliate Shannon diversity	*F* = 0.1841, *p* = 0.6779	*F* = 0.4344, *p* = 0.5263	*F* = 8.9153, *p* = 0.0015**
18S	Zooplankton Shannon diversity	*F* = 0.7842, *p* = 0.3989	*F* = 0.5811, *p* = 0.4654	*F* = 4.1071, *p* = 0.0305*
COI	Zooplankton Shannon diversity	*F* = 0.9409, *p* = 0.3574	*F* = 0.0101, *p* = 0.9221	*F* = 10.3405, *p* = 0.0007***
COI	Phytoplankton Shannon diversity	*F* = 2.3246, *p* = 0.1617	*F* = 0.1468, *p* = 0.7105	*F* = 20.4191, *p* < 0.0001***
Microscopy	Zooplankton Shannon diversity	*F* = 1.0161, *p* = 0.3398	*F* = 1.0460, *p* = 0.3331	*F* = 2.4802, *p* = 0.1068
Microscopy	Phytoplankton Shannon diversity	*F* = 6.3972, *p* = 0.0323*	*F* = 2.8867, *p* = 0.1235	*F* = 2.6761, *p* = 0.0911
Microscopy	Ciliate Shannon diversity	*F* = 0.2093, *p* = 0.6582	*F* = 3.9986, *p* = 0.0766	*F* = 1.5718, *p* = 0.2301
18S	Zooplankton Evenness	*F* = 0.9941, *p* = 0.3448	*F* = 0.00075, *p* = 0.9787	*F* = 6.6690, *p* = 0.0054**
18S	Phytoplankton Evenness	*F* = 0.6392, *p* = 0.4446	*F* = 0.2033, *p* = 0.6627	*F* = 0.7716, *p* = 0.4744
18S	Ciliate Evenness	*F* = 0.3271, *p* = 0.5814	*F* = 0.1362, *p* = 0.7207	*F* = 1.7804, *p* = 0.1956
COI	Zooplankton Evenness	*F* = 1.8424, *p* = 0.2077	*F* = 0.0130, *p* = 0.9116	*F* = 9.0956, *p* = 0.0013**
COI	Phytoplankton Evenness	*F* = 2.7221, *p* = 0.1334	*F* = 0.2505, *p* = 0.6288	*F* = 14.3389, *p* = 0.0001***
Microscopy	Zooplankton Evenness	*F* = 1.0286, *p* = 0.3370	*F* = 0.6605, *p* = 0.4374	*F* = 2.4335, *p* = 0.1110
Microscopy	Phytoplankton Evenness	*F* = 0.0275, *p* = 0.8719	*F* = 5.0785, *p* = 0.0507	*F* = 1.4398, *p* = 0.2585
Microscopy	Ciliate Evenness	*F* = 0.0000, *p* = 0.9965	*F* = 0.2630, *p* = 0.6201	*F* = 4.5120, *p* = 0.0228*

*Note:* Significance levels are indicated as *p < 0.05, **p < 0.01 and ***p < 0.001.

The proportion of microscopy‐identified species and genera that were also recovered from reference databases and metabarcoding data varied across taxonomic groups and markers (Figure S7). Zooplankton showed the highest overlap, with 57% of species and 83% of genera detected by COI and 57% of species and 74% of genera recovered by 18S. Phytoplankton also showed considerable overlap with 18S (53% species, 64% genera), but lower congruence with COI (17% species, 37% genera). Ciliates had the lowest overlap overall, with only 25% of species and 44% of genera matching 18S ASVs (COI does not amplify ciliates). Across all groups, matches based on exact sequence identity (100%) were lower than those based on taxonomic names. The 18S marker provided broader taxonomic coverage, especially for phytoplankton and ciliates, while COI achieved higher genus‐level recovery for zooplankton.

### 3.2 Taxon Level

#### 3.2.1 Microscopy

**FIGURE 4:**
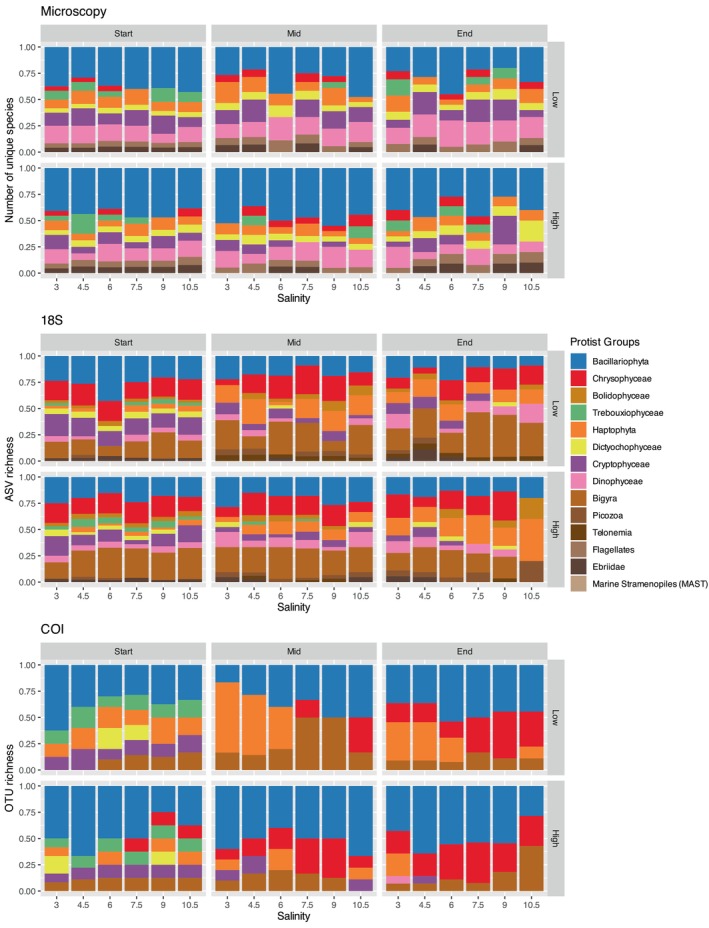
Protist community composition and richness based on microscopy, 18S and COI datasets across salinity levels, temperature treatments (Low, High) and time points (Start, Mid, End).

**FIGURE 5:**
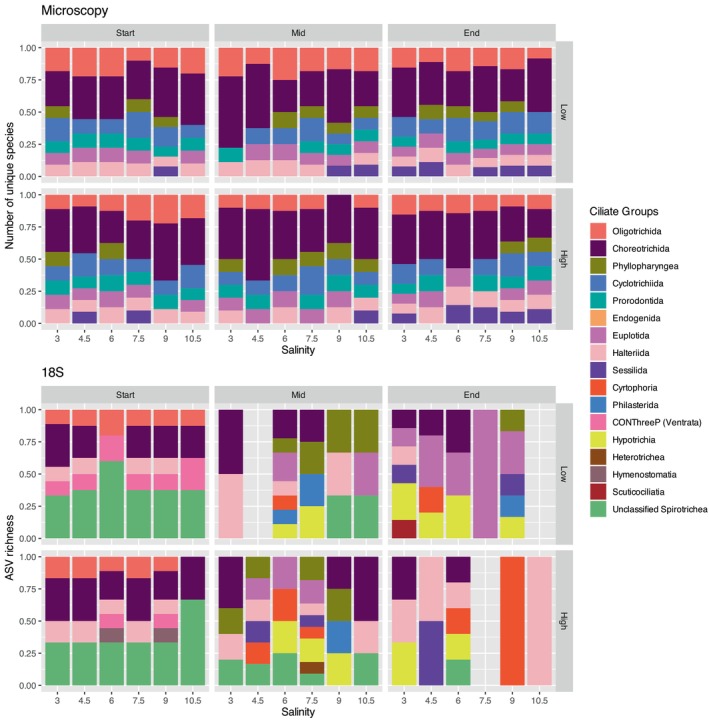
Ciliate community composition and richness based on microscopy and 18S datasets across salinity levels, temperature treatments (Low, High) and time points (Start, Mid, End).

**FIGURE 6:**
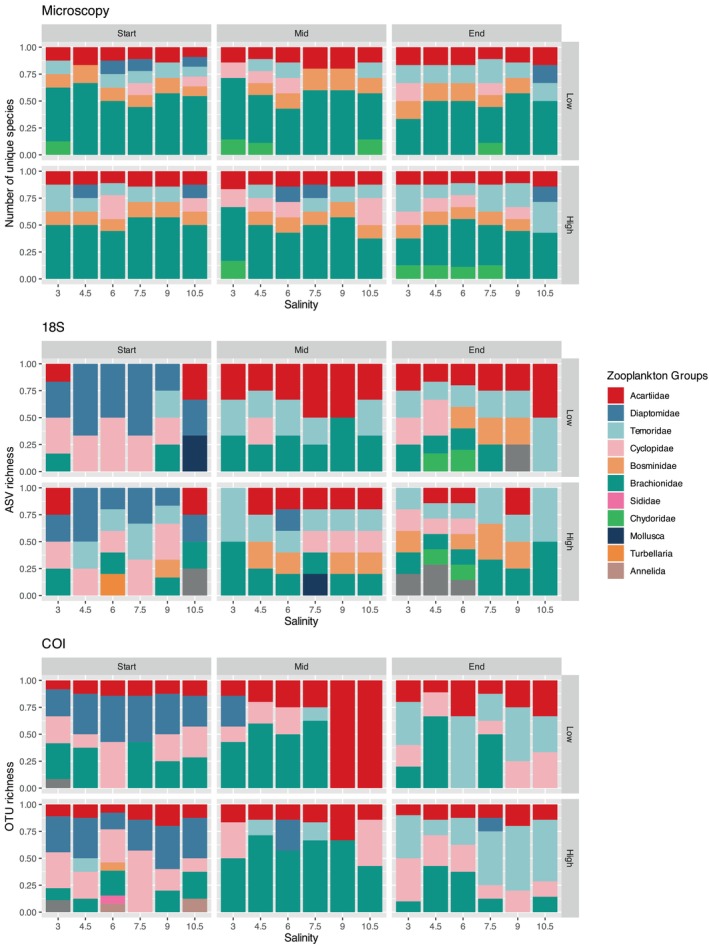
Mesozooplankton community composition and richness based on microscopy, 18S and COI datasets across salinity levels, temperature treatments (Low, High) and time points (Start, Mid, End).

A total of 146 plankton taxa were detected using microscopy, including 22 zooplankton taxa, 14 ciliate taxa and 114 phytoplankton taxa (Figures 4, 5, 6). Rotifers and copepod groups were significantly influenced by salinity and temperature (Table 2). Rotifers were more abundant at lower salinities. The rotifer genus *Brachionus* was more abundant at higher temperatures, while *Keratella quadrata* was more abundant in low salinities. Copepods and copepod nauplii were significantly more abundant at higher salinities, but species‐specific responses differed, as the genus *Mesocyclops* was more abundant in lower salinities. The copepod genus *Acartia* exhibited a significant interaction between temperature and salinity, showing its highest abundance at both high temperatures and high salinities. For ciliates, *Strombidium*, *Laboea* and *Telonema subtile* were more abundant at lower temperatures, while *Vorticella* was more abundant at higher salinities. *Strobilidium* exhibited a significant temperature–salinity interaction: high salinity did not directly increase its abundance, but it mitigated the negative impact of high temperatures. In contrast, at low salinity, the negative effect of high temperature was more pronounced, leading to lower abundance (Table 2). Within phytoplankton, Chlorophyta, *Monoraphidium contortum* , *Pyramimonas*, Cryptophyta (including *Teleaulax* and *Telonema subtile*), *Cyclotella choctawhatcheeana*, Peridiniales and unidentified flagellates (size class 2–5 μm) were all more abundant at lower temperatures. Phytoplankton (overall abundance) and cyanobacteria were more abundant in lower salinities (Table 2).

#### 3.2.2 Metabarcoding

A total of 427 ASVs were assigned using the 18S marker, while 369 OTUs were assigned using COI. The 18S marker primarily identified ciliates, copepods, cladocerans, rotifers and flagellates, whereas COI mainly detected diatoms, rotifers and copepods (Figures 4, 5, 6). A portion of OTUs/ASVs remained unclassified beyond the Eukaryota level, accounting for 5.5% in 18S and 10% in COI. The copepod species *Acartia tonsa* was more abundant in warmer temperatures. Among ciliates, CONThreeP (Ventrata) was more abundant in higher temperatures. For phytoplankton, *Skeletonema subsalsum* , Chromulinales, *Pedospumella sinomuralis* and Trebouxiophyceae were more abundant in lower temperatures, while *Chrysochromulina parva* , Pyramimonadales and *Telonemia* were more abundant in higher ones. Among other groups detected by metabarcoding, the macroalgae *Nemacystus decipiens* was more abundant in higher temperatures. For salinity effects, Anthoathecata and Diptera were more abundant in higher salinities. There was a significant interaction effect of temperature and salinity found for the ameboflagellate order Filosa‐Thecofilosea_X, which was more abundant in low temperatures and high salinities (Table 5).

**TABLE 5:** Differential abundance model results for metabarcoding data for plankton group abundances with warming and salinity. Only results for temperature and salinity and their interaction are shown for clarity.

Method	Rank	Response	Temperature	Salinity	Salinity × temperature
18S	Class	CONThreeP	LFC = 2.799798, *p* = 0.01921608*		
18S	Class	Pyramimonadophyceae	LFC = 3.115376, *p* = 0.01921608*		
18S	Class	Telonemia_XX	LFC = 4.456178, *p* = 0.04783957*		
18S	Class	Trebouxiophyceae	LFC = −2.018207, *p* = 0.04654618*		
18S	Order	CONThreeP_X	LFC = 3.097158, *p* = 0.006063964**		
18S	Order	Pyramimonadales	LFC = 3.413529, *p* = 0.006063964**		
18S	Order	Filosa‐Thecofilosea_X			LFC = 3.360713, *p* = 0.03744625*
18S	Species	*Skeletonema subsalsum*	LFC = −2.927553, *p* = 0.01483334*		
COI	Class	Chrysophyceae	LFC = −3.318722, *p* = 0.009261685*		
COI	Class	Hexanauplia	LFC = 1.511157, *p* = 0.009473052**		
COI	Order	Calanoida	LFC = 1.9443091, *p* = 0.003187944**		
COI	Order	Chromulinales	LFC = −3.1200282, *p* = 0.010677270*		
COI	Order	Anthoathecata		LFC = 0.9906546, *p* = 0.039570896*	
COI	Order	Diptera		LFC = 1.3033736, *p* = 0.039570896*	
COI	Genus	*Acartia*	LFC = 1.911603, *p* = 0.03629750*		
COI	Genus	*Nemacystus*	LFC = 2.093648, *p* = 0.02035724*		
COI	Genus	*Pedospumella*	LFC = −3.030110, *p* = 0.02035724*		
COI	Species	*Acartia tonsa*	LFC = 1.958187, *p* = 0.02209570*		
COI	Species	*Chrysochromulina parva*	LFC = 5.535857, *p* = 0.03276341*		
COI	Species	*Nemacystus decipiens*	LFC = 2.147187, *p* = 0.01639483*		
COI	Species	*Pedospumella sinomuralis*	LFC = −2.978491, *p* = 0.01639483*		

*Note:* Significance levels are indicated as *p < 0.05, **p < 0.01 and ***p < 0.001.

Abbreviation: LFC, Log Fold Change.

## 4 Discussion

### 4.1 Overview of Findings

The aim of this study was to investigate how climate‐driven changes in salinity and temperature affect plankton communities using both microscopy and DNA metabarcoding. We hypothesised that temperature and salinity would have distinct effects on different plankton groups, with warming favouring smaller, fast‐growing taxa and salinity filtering community composition based more on species' tolerances. We also expected interactive effects between these drivers, as well as clear, directional changes in diversity and composition along both environmental gradients.

Our findings partially support these expectations. Warming had a strong effect on zooplankton richness, but not necessarily on their total abundance, suggesting indirect or trait‐mediated responses rather than uniform population increases. This pattern may be explained by an indirect warming effect: within our tested range, higher temperatures likely elevated zooplankton metabolic rates and diversity, which in turn increased grazing pressure and contributed to a decline in phytoplankton. This aligns with previous findings that warming can intensify top‐down control in plankton food webs (Rose and Caron 2007; O'Connor et al. 2012). Salinity, on the other hand, significantly influenced community composition, particularly within zooplankton, with rotifers more abundant at lower salinities and copepods dominating at higher salinities. This indicates that salinity acted as a strong environmental filter, particularly for higher trophic levels. Phytoplankton diversity also declined with increasing salinity, reflecting both reduced taxonomic richness and changes in community composition. These patterns were likely driven by species‐specific physiological tolerances to osmotic stress and ionic conditions (Telesh and Khlebovich 2010) and may have been further shaped by grazing from salinity‐favoured zooplankton such as copepods. At the taxon level, phytoplankton and ciliates (lower trophic levels) were more responsive to warming, while zooplankton (higher trophic levels) showed stronger responses to salinity—contrary to our original prediction that warming would have the most pronounced impact on large, heterotrophic taxa. This complexity highlights the importance of species‐specific traits and interactive effects between stressors in shaping community responses.

In addition, our comparison of methods showed that metabarcoding and microscopy were complementary rather than interchangeable. Metabarcoding detected a broader range of taxa, including many cryptic or small‐sized organisms, but revealed fewer individual taxon‐level responses to treatments compared to microscopy. In contrast, microscopy provided better quantitative resolution, particularly for groups like rotifers and ciliates. Below, we first discuss ecological findings in detail, followed by a critical evaluation of methodological performance and implications for future studies.

### 4.2 Salinity Effects on Plankton Diversity

Lower salinities supported a richer, more diverse (Shannon) and overall more abundant phytoplankton community, suggesting that freshwater or salinity‐tolerant species were able to thrive. This is possibly because true marine taxa could not establish at the maximum salinity of 10.5 PSU and consequently, freshwater species dominated, while the absence of fully marine conditions excluded marine specialists. This finding aligns with the Remane concept (Remane 1934), which predicts a richness minimum at intermediate salinities (roughly 7–10 PSU) due to the limited tolerance of both freshwater and marine organisms in brackish conditions, compounded by the scarcity of true brackish specialists (Paavola et al. 2005; Elliott and Whitfield 2011). Indeed, studies in the Baltic Sea have documented higher diversity at lower (< 7 PSU) and higher (> 10 PSU) salinities, with a dip in richness around 7 to 10 PSU (Pawlak et al. 2009; Olli et al. 2023). Because this experiment did not exceed 10.5 PSU, it likely favoured freshwater taxa, resulting in greater phytoplankton richness at lower salinities and no clear opportunity for marine species to become dominant. It is therefore important to note that patterns of diversity may differ at higher salinities beyond our experimental scope, particularly in more marine‐dominated regions (e.g., > 15 PSU) where marine specialists would be more competitive. This reflects broader seasonal and biogeographic variability in plankton communities across the Baltic Sea (Serandour et al. 2024), which should be considered when extrapolating our findings. Lower salinity significantly increased cyanobacteria, indicating that potential freshening may favour their proliferation, as seen in studies elsewhere (Rosen et al. 2018). Since cyanobacteria drive harmful algal blooms in the Baltic Sea, scenarios predicting declining salinity could further intensify these already frequent summer occurrences.

### 4.3 Salinity Effects at the Taxon‐Level

Effects of salinity at the taxon level were mainly apparent on grazers in microscopy data. Rotifers were more abundant in lower salinities, while copepods and copepod nauplii were more abundant in higher salinities. However, species‐specific responses differed. The rotifer, *Keratella quadrata*, and copepod, *Mesocyclops*, both favoured lower salinities, whereas the copepod *Acartia* exhibited a significant interaction effect, reaching its highest abundance at both high temperatures and high salinities. The ciliate *Vorticella* was also more abundant in higher salinities. In previous experiments, we found a shift to a rotifer‐dominated community in low salinities of the Baltic Sea, potentially corresponding to a reduction of copepods, reducing predation and competition (Brandl 2005; Hall and Lewandowska 2022). Additional studies have also shown that rotifers make up the most dominant proportion of the zooplankton community in freshwater environments (Medeiros et al. 2010) and that rotifers have been increasing in the Baltic Sea over the past 50 years (Jansson et al. 2020). The copepod *Acartia tonsa* is commonly attributed as being the ‘marine’ genus of copepod in the Baltic Sea tolerating a wide range of salinities (Svetlichny and Hubareva 2014), with an optimum between 10 and 20 PSU (Cervetto et al. 1999; Calliari et al. 2006). This is compared to *Mesocyclops*, which is generally a more freshwater species and cannot stand salinities of over 7.5 PSU (Leppäkoski et al. 2002; Dela Paz et al. 2016), hence its prevalence in low salinities. The ciliate, *Vorticella*, is a genus of marine ciliate and has been shown to withstand high salinity concentrations (Salvadó et al. 2001) and is also an epibiont of the abundant genus of diatom, *Chaetoceros*, during this experiment (Gómez et al. 2018; Nanajkar et al. 2019). Overall, these responses suggest a more ‘classic’ marine food web structure prevails under higher salinities, where top‐down control of the system is maintained by abundance of large predatory zooplankton like marine copepods, compared to lower salinities, which resulted in a shift towards smaller zooplankton such as rotifers and freshwater species.

Microscopy detected significant salinity‐driven changes in individual taxa within zooplankton and ciliate communities, as supported by linear model results (Tables 2 and 4). In contrast, metabarcoding did not capture similar patterns in these target plankton groups, with LinDA analysis instead identifying changes in non‐target taxa such as Anthoathecata (Cnidaria) and Diptera at higher salinities (Table 5). One notable exception was the order Filosa‐Thecofilosea_X, which increased in high‐salinity and low‐temperature conditions. As predominantly algivorous protists (Dumack et al. 2022), their higher abundance may be linked to the increase in phytoplankton at lower temperatures found in the experiment, suggesting a bottom‐up effect where greater food availability supports their population growth. No significant salinity responses were detected for phytoplankton, ciliates or copepods. The absence of significant trends for rotifers and ciliates with metabarcoding contrasts with microscopy, suggesting potential biases in metabarcoding related to DNA copy number, primer specificity, or amplification efficiency as both groups were somewhat underrepresented in metabarcoding data. These discrepancies highlight the limitations of metabarcoding in detecting quantitative community shifts and suggest that integrating complementary approaches, such as quantitative PCR (qPCR), could improve abundance estimates and enhance detection of finer‐scale ecological responses to salinity changes.

### 4.4 Warming Effects on Plankton Diversity

Warming led to an overall more even and diverse community composition, as well as a higher species richness of zooplankton in the experiment, reflected in both microscopy and metabarcoding data. This could be due to dominant plankton species that are locally adapted to ambient Baltic Sea temperature and might lose their competitive advantage over warm water species. As their dominance declines, a broader range of plankton species can coexist, contributing to a more even community composition. Warmer temperatures can also enhance nutrient cycling and primary productivity in aquatic ecosystems, possibly leading to the increased abundance of certain protist groups in the experiment (e.g., *Chrysochromulina parva*, Pyramimonadales and *Telonemia*). This can result in higher food availability for grazers, reducing resource limitation for a wider range of species to coexist and hence supporting the increased zooplankton diversity we found here. When resources are more abundant, communities may exhibit a more even and diverse distribution because of fewer constraints on the coexistence of different competitors. This has also been shown elsewhere with warming leading to increased diversity of plankton (Ibarbalz et al. 2019) as well as in community evenness. Yvon‐Durocher et al. (2015) found that warming led to an increase in phytoplankton species richness and suggested this was due to zooplankton consumers exhibiting stronger metabolism‐mediated top‐down control on phytoplankton communities. This led to the dominance of grazing‐resistant phytoplankton species, due to their larger cell size and/or colonial or filamentous growth form. These trophic interactions led to an immigration–extinction equilibrium, where consumer–resource body size ratios converge towards those that are most stable. With the predicted warming of the Baltic Sea, the observed increase in both overall plankton diversity and zooplankton species richness suggests that, at least in the short term, any cascading effects on ecosystem stability and trophic interactions may be counterbalanced by increased species coexistence and resource availability. However, long‐term implications remain uncertain, as shifts in community structure could alter competitive dynamics, energy transfer and food web stability over time.

### 4.5 Warming Effects at the Taxon‐Level

Microscopy data showed that warming had limited effects on mesozooplankton taxa, primarily influencing rotifers, while ciliates and phytoplankton exhibited a stronger response. The rotifer genus *Brachionus* was more abundant in warmer temperatures. As the second most abundant rotifer genus after *Keratella* in the experiment, *Brachionus* has a higher optimum temperature range (Galkovskaja 1987), which may have given it a competitive advantage over *Keratella* in warmer conditions. On the other hand, metabarcoding results revealed that *Acartia tonsa* was more abundant in warmer temperatures, while microscopy suggested its response to warming was mediated by salinity. The observed increase in *Acartia tonsa* under warming aligns with the hypothesis that temperature‐driven metabolic acceleration may enhance reproductive rates and recruitment in copepod species such as *Acartia* (Sullivan and McManus 1986; Choi et al. 2021) as well as benefit early life stages such as nauplii (Richardson 2008; Daufresne et al. 2009). Other studies have shown that higher salinity may improve *Acartia* tolerance to warming by enhancing oxidative status and reproductive performance (Von Weissenberg et al. 2022). Thus, under future climate scenarios for the Baltic Sea, areas experiencing increased salinity might exhibit greater resilience to warming, whereas regions undergoing freshening could potentially see reduced resilience. However, microscopy data showed that copepod nauplii responded to salinity rather than temperature, suggesting that salinity could play a more critical role in reproduction.

Ciliates also responded to warming, and the abundance of mixotrophic ciliates *Strombidium* and *Laboea* reduced with warming in microscopy data. Even though *Strombidium* has been found to tolerate a wide range of temperatures (Lee et al. 2012; Montagnes et al. 2008), its decline suggests that warming may have indirectly affected its survival. The decline of *Strombidium* could have broader implications for cyanobacterial blooms, as it is an efficient grazer of cyanobacteria and heterotrophic bacteria (Bernard and Rassoulzadegan 1990) potentially leading to an increase in bloom frequency and intensity in the Baltic Sea. The thermal tolerance of *Laboea* is not well studied, but as a mixotrophic ciliate, it likely relies on both photosynthesis and heterotrophic feeding, making it susceptible to changes in nutrient availability and prey abundance. *Strobilidium* showed a significant interaction between temperature and salinity: warming reduced its abundance, but the decline was much stronger under low salinity than under high salinity. This suggests that high salinity may buffer the negative effects of warming, possibly by maintaining osmotic balance or reducing physiological stress. In contrast, low salinity may increase cellular stress under warming, making *Strobilidium* less able to tolerate elevated temperatures. This indicates that while *Strobilidium* may have some capacity to withstand higher temperatures, it is primarily vulnerable to warming, particularly in areas experiencing freshening. This interactive effect highlights the compounding impact of multiple stressors, suggesting that the combined effects of warming and freshening may disrupt populations more severely than either factor alone. Understanding such interactions is crucial for predicting how climate change will shape plankton communities and ecosystem dynamics in the future. Ciliate response may have also been affected indirectly through a reduction in their food source found in the experiment in warmer temperatures: Chlorophyta and Haptophyta (genus: *Prymnesiales*). Interestingly, since phytoplankton abundance also declined under warming, it is possible that mixotrophic ciliates were impacted not only by prey limitation but also by reduced autotrophic efficiency. If the same environmental factors that hindered phytoplankton photosynthesis—such as reduced nutrient availability or temperature‐driven metabolic stress—also affected the symbiotic algae's photosynthetic mechanisms in mixotrophic ciliates, then *Strombidium* and *Laboea* may have struggled to meet their energy demands.

Furthermore, metabarcoding results also indicated an increase in heterotrophic taxa with warming, including heterotrophic ciliates *CONThreeP (Ventrata)* and heterotrophic flagellates *Telonemia*, suggesting that bacterivory and predation on small protists (Bråte et al. 2010) may become more dominant pathways under warming. These groups may have thrived due to increased bacterial production or picoplankton biomass, potentially suppressing cyanobacteria increases despite predictions of their proliferation under warming. This shift from mixotrophy to heterotrophy may indicate a fundamental restructuring of microbial food webs in response to climate‐driven temperature increases. However, the lack of a clear cyanobacterial response to warming, along with the absence of mixotrophic ciliate shifts in metabarcoding data, suggests temperature tolerance as well as trophic strategy drive ciliate community shifts.

Both microscopy and metabarcoding results revealed temperature‐driven shifts in phytoplankton communities, though the patterns differed between methods. Microscopy results suggested an overall reduction in phytoplankton abundance across multiple groups in response to warming. Specifically, there was a decline across multiple phytoplankton groups and species, including Chlorophyta, Cryptophyta, Haptophyta and unidentified flagellates (2–5 μm). These results were also backed up by fluorescence data (representing chlorophyll‐a content) which also showed an overall decline in warmer temperatures. Metabarcoding data also provided support for this shift, detecting reductions in the diatom *Skeletonema subsalsum*, Chromulinales, *Pedospumella sinomuralis* and Trebouxiophyceae. This suggests that larger, more temperature‐sensitive or autotrophic taxa were particularly affected, while mixotrophic and heterotrophic taxa may have been more resilient. Several of the phytoplankton taxa that declined under warming, including diatoms, Chlorophyta and Cryptophyta, are characterized by relatively high chlorophyll‐a content per cell (Hillebrand et al. 1999; Jakobsen and Markager 2016). Their loss likely contributed significantly to the reduction in fluorescence observed in the warmer treatments. In contrast, cooler treatments sustained higher and more variable fluorescence levels, likely due to the continued presence and episodic growth of these pigment‐rich groups. This variability may reflect dynamic phytoplankton turnover driven by rapid growth and mortality, shifts in community composition, or changes in the photosynthetic performance of different species, followed by sharp declines—potentially linked to increased grazing pressure. The latter is supported by the observed increase in zooplankton richness, particularly copepods, and a shift towards heterotrophic ciliate taxa under warming, suggesting stronger top‐down control in warmer conditions. The decline in fluorescence and phytoplankton abundance aligns with global trends of reduced biomass under warming (Behrenfeld et al. 2006; Doney 2006; Boyce et al. 2010) and may be linked to changes in stratification, nutrient availability and direct impacts on photosynthetic efficiency, enzymatic activity and metabolic rates. These changes likely disadvantage strictly autotrophic taxa while favouring mixotrophic and heterotrophic groups that can supplement energy demands through alternative feeding strategies (Edwards 2019). These effects are likely to propagate to higher trophic levels, as warming has also been linked to reduced phytoplankton elemental density, limiting energy transfer to grazers (Krause and Lomas 2020). In addition, the timing of the experiment may have influenced these results, as nutrient availability is typically lower in the Baltic Sea during late summer, making autotrophic taxa less competitive against mixotrophic taxa (Egge 1998). While flagellates were expected to benefit from warming due to their motility and metabolic flexibility (Egge 1998), their decline suggests that other factors, such as increased predation from higher trophic levels, may have counteracted their ability to thrive. Higher fluorescence found in cooler treatments may indicate better chlorophyll‐*a* retention and photosynthetic efficiency, such as enhanced light‐harvesting capacity (Mai et al. 2021). This reinforces the idea that warming reduces autotrophic dominance while favouring more flexible survival strategies, potentially altering primary production in the system (Cvjetinovic et al. 2020).

Despite the general decline in phytoplankton under warming, some taxa did increase in response to higher temperatures. Metabarcoding detected a higher abundance of haptophytes ( *Chrysochromulina parva* ), green algae (Pyramimonadales) and *Telonemia* in warmer conditions. These taxa may have benefited from higher metabolic rates, mixotrophic feeding capabilities, or improved nutrient uptake efficiencies under nutrient‐limited conditions, suggesting that warming selectively favoured phytoplankton with more flexible survival strategies. The increased abundance of mixotrophic taxa like *Chrysochromulina parva* (Trochine et al. 2015) supports the idea that warming may have favoured phytoplankton capable of supplementing photosynthesis with heterotrophic feeding, giving them an advantage over strictly autotrophic competitors. *Chrysochromulina* is a bloom‐forming haptophyte genus that can be toxic to fish (Edvardsen et al. 1998), suggesting that areas experiencing freshening in the Baltic Sea may promote its proliferation, with possible detrimental effects on higher trophic levels (Telesh and Skarlato 2022; Schmidt 2002). Other bloom‐forming groups like cyanobacteria, expected to increase with warming (Paerl and Huisman 2008; Boyd et al. 2014), showed no response to temperature in this experiment. This may be due to the seasonal timing of the study, as cyanobacterial blooms typically peak in mid to late summer (July–August) when temperatures are highest, and nutrient conditions favour bloom formation (Kahru and Elmgren 2014). By September, when this experiment was conducted, populations may have already declined naturally, potentially explaining the lack of response.

Shifts in phytoplankton abundance may not be solely due to direct temperature effects but also increased grazing pressure under warming. Higher temperatures elevate zooplankton metabolism, leading to increased energy demands and stronger top‐down control on phytoplankton (Aberle et al. 2007). In well‐mixed, nutrient‐rich seas like the Baltic, copepod predation on diatoms can significantly reduce their biomass (Lewandowska et al. 2014) and the fact we found higher abundance of *Acartia tonsa* in warmer temperatures could support this. Due to the increased zooplankton richness found in warmer temperatures, this could have also triggered stronger metabolism‐mediated, top‐down control from zooplankton on phytoplankton communities through a more diverse consumer community better able to reduce producers (Rose et al. 2009).

### 4.6 Comparison of Metabarcoding and Microscopy Results and Limitations

The differences between microscopy and metabarcoding findings highlight the complexity of phytoplankton responses to warming. Both methods showed a decline in phytoplankton groups, but metabarcoding revealed species‐specific increases, suggesting that warming did not simply reduce overall phytoplankton abundance but instead drove a community shift. Despite these differences, both methods indicate that warming reduces larger, cold‐adapted phytoplankton, particularly diatoms and flagellates, while favouring smaller, thermally tolerant or mixotrophic taxa. This shift could disrupt nutrient cycling, trophic interactions, and carbon flow, as smaller phytoplankton provide less food for larger grazers, potentially reshaping Baltic Sea plankton dynamics under future climate scenarios.

We observed good overlap in species detection between metabarcoding and microscopy when identifying plankton taxa. Metabarcoding detected a higher number of taxa, but of those taxa that were detected using microscopy, these were also present in the metabarcoding data, suggesting minimal mismatches in the molecular technique. Overall, richness‐based metrics from metabarcoding tended to align more closely with microscopy results. In contrast, metabarcoding relative abundance data (Figures S1–S3) were often dominated by a few high‐biomass or high‐copy‐number taxa, potentially obscuring shifts in community composition among less abundant but ecologically relevant groups. Despite both approaches consistently identifying major taxonomic groups, their responses to the experimental treatments varied. The microscopy‐based models primarily detected salinity‐driven shifts in zooplankton and ciliates but also revealed temperature‐driven changes, particularly in ciliates and phytoplankton. In contrast, metabarcoding‐based models revealed temperature responses across fewer taxa, particularly phytoplankton and copepods. *Telonemia* (metabarcoding) and *Telonema* (microscopy) as well as Pyramimonadales (metabarcoding) and *Pyramimonas* (microscopy) showed conflicting trends, with microscopy detecting a reduction in warmer temperatures, while metabarcoding suggested an increase. These discrepancies likely stem from methodological differences. Microscopy relies on direct cell counts, which may underestimate smaller or morphologically indistinct taxa, while metabarcoding captures genetic material from all life stages, including resting cysts and degraded cells. This may explain why some taxa appeared stable or even increased, despite microscopy showing a decline.

Microscopy and metabarcoding often operate at different taxonomic and quantitative resolutions, which can lead to discrepancies in ecological interpretation. Metabarcoding detected a higher number of taxa overall, especially small or cryptic groups such as protists that are often overlooked in microscopy. In contrast, microscopy was more effective for quantifying rotifers and ciliates, which were underrepresented in metabarcoding results. For example, the COI marker failed to detect rotifers in some treatments despite their high abundance in microscopy (e.g., Brachionidae), while the 18S marker classified only 43% of ciliates beyond the class level (Kulaš et al. 2021). These differences in primer performance likely contributed to the weaker salinity responses observed in the metabarcoding data, as key community‐level changes may have been obscured by incomplete amplification or low taxonomic resolution (Blanco‐Bercial 2020; van der Loos and Nijland 2021). In addition, the statistical approaches differed slightly: LinDA, used for metabarcoding, is designed for compositional data and applies conservative corrections to limit false positives, which may reduce sensitivity to subtle taxon‐level changes. In contrast, the linear mixed models used for microscopy rely on absolute counts and may better capture gradual abundance shifts. These analytical differences, together with the inherent contrast between absolute (microscopy) and relative (metabarcoding) abundance data, likely contributed to variation in detection power between methods. For instance, copepods appeared overrepresented in metabarcoding, likely due to higher DNA content, while in microscopy their nauplii were only identified to developmental stage. This mismatch in resolution may have further contributed to differences in interpretation between the two methods. The dominance of *Eurytemora affinis* in 18S reads and conversely *Acartia tonsa* in COI (Figure S3), despite similar abundances in microscopy, further supports the influence of marker‐specific amplification biases.

We did have some concern that DNA persistence could obscure real‐time community changes. However, in our case, the weekly sampling intervals likely allowed sufficient time for environmental DNA from previously present organisms to degrade. Therefore, the metabarcoding results are expected to reflect the active community at the time of sampling rather than legacy signals. For even finer temporal resolution, future studies could consider targeting RNA, which degrades more rapidly and provides a better representation of metabolically active organisms (Pochon et al. 2017). In addition, some limitations stem from the experimental setup. Our use of a continuous salinity gradient allowed us to assess responses across a realistic environmental range, but the lack of replication at individual salinity levels may have reduced statistical power to detect more subtle or variable responses in models. Moreover, since our mesocosm conditions did not extend beyond 10.5 PSU, the observed shifts likely reflect the responses of freshwater‐ to brackish‐adapted taxa. We therefore caution against generalising these outcomes to more marine assemblages. Despite these limitations, both 18S and COI detected clear warming effects, and these patterns broadly aligned with microscopy‐based observations. To further improve the interpretation of metabarcoding data, future studies could incorporate mock communities (Shelton et al. 2023) to help address these biases by estimating starting DNA proportions rather than relying on post hoc transformations.

Metabarcoding also offers advantages for biodiversity assessments and invasive species detection, where quantification is less critical. It appears more responsive to overall community composition changes over time, highlighting its potential for time‐series analysis (Suter et al. 2021; Bucklin et al. 2022). However, integrating multiple molecular markers and expanding reference databases would improve resolution, particularly for groups affected by primer bias. Environmental factors such as temperature and salinity can also influence DNA degradation rates (Collins et al. 2018; Qian et al. 2022), further complicating eDNA‐based assessments. Future studies should also consider RNA‐based approaches to mitigate DNA degradation biases (Miyata et al. 2022; Kagzi et al. 2023) and refine experimental setups to enhance replication and primer selection. A complementary approach leveraging microscopy's quantitative accuracy with metabarcoding's taxonomic breadth will likely improve plankton monitoring and ecological assessments (Laakmann et al. 2020).

## 5 Conclusion

We demonstrate that predicted changes in salinity and warming in the Baltic Sea will significantly alter plankton community structures. Specifically, under scenarios of decreasing salinity, we anticipate a reduction in more complex, large zooplankton like marine species copepods and the dominance of smaller‐sized freshwater zooplankton groups, such as rotifers. We also expect a shift to smaller phytoplankton, mixotrophic and heterotrophic protists, and an increased abundance of bloom‐forming freshwater algae and cyanobacteria. These community shifts are not only driven by direct effects of temperature and salinity but also by their interactions, as seen in cases where salinity buffered temperature effects on some taxa, while in others, warming intensified salinity‐driven declines. This highlights the need to consider multiple stressors simultaneously when predicting climate change impacts on aquatic ecosystems. Our study provides valuable insights into the application of metabarcoding in mesocosm experiments, highlighting both its advantages and limitations in detecting shifts in plankton community composition under controlled environmental conditions. We find that metabarcoding was complimentary to microscopy, rather than acting as a replacement. Metabarcoding was able to access a wider diversity of taxa we would not have been able to identify with microscopy. Both methods revealed responses to salinity and warming, but with limited agreement in how taxa responded to these treatments, highlighting the importance of using complementary approaches. Our findings reinforce the growing recognition of metabarcoding as a powerful tool for assessing climate‐driven changes in plankton communities, particularly in response to shifts in salinity and temperature. To improve its accuracy and quantitative reliability, future research should focus on refining bioinformatics pipelines and molecular techniques. By advancing the use of metabarcoding in mesocosm studies, we contribute to a more robust methodology for understanding environmental changes in plankton food webs and aquatic ecosystems.

## Author Contributions

**Clio Abbie Marjorie Hall:** conceptualization (lead), data curation (lead), formal analysis (lead), funding acquisition (lead), investigation (lead), methodology (lead), project administration (lead), resources (lead), validation (lead), visualization (lead), writing – original draft (lead), writing – review and editing (lead). **Nicolas Henry:** data curation (equal), formal analysis (equal), methodology (equal), software (equal), writing – review and editing (equal). **Oriol Canals:** data curation (equal), investigation (equal), methodology (equal), writing – review and editing (equal). **Gianina Consing:** formal analysis (supporting), investigation (supporting), writing – review and editing (supporting). **Naiara Rodríguez‐Ezpeleta:** formal analysis (supporting), methodology (supporting), writing – review and editing (supporting). **Aleksandra M. Lewandowska:** conceptualization (equal), funding acquisition (supporting), investigation (supporting), methodology (supporting), project administration (equal), supervision (equal), writing – review and editing (supporting).

## Conflicts of Interest

The authors declare no conflicts of interest.

## Supporting information


**Figure S1:** ece372125‐sup‐0001‐FiguresS1‐S8.pdf.

## Data Availability

Raw sequencing data have been deposited in the European Nucleotide Archive (ENA) under accession number PRJEB79753 and are accessible at: https://www.ebi.ac.uk/ena/browser/view/PRJEB79753. All processed data and analysis scripts supporting the findings of this study are available via the Dryad Digital Repository at: https://doi.org/10.5061/dryad.bvq83bkkq. Details about bioinformatic workflows used in this study are available at: https://gitlab.com/tvarminne‐metabarcoding/mesocosm‐18s‐bioinfo and https://gitlab.com/tvarminne‐metabarcoding/mesocosm‐coi‐bioinfo.
