# Supplementary material for: DNA Metabarcoding as a Tool to Study Plankton Responses to Warming and Salinity Change in Mesocosms
Source: Ecol Evol. 2025 Sep 15;15(9):e72125. doi: 10.1002/ece3.72125 (PMC12434319; doi:10.1002/ece3.72125)
Supplement: Supplementary file 1 — Figure S1: ece372125‐sup‐0001‐FiguresS1‐S8.pdf. [file ECE3-15-e72125-s001.pdf]

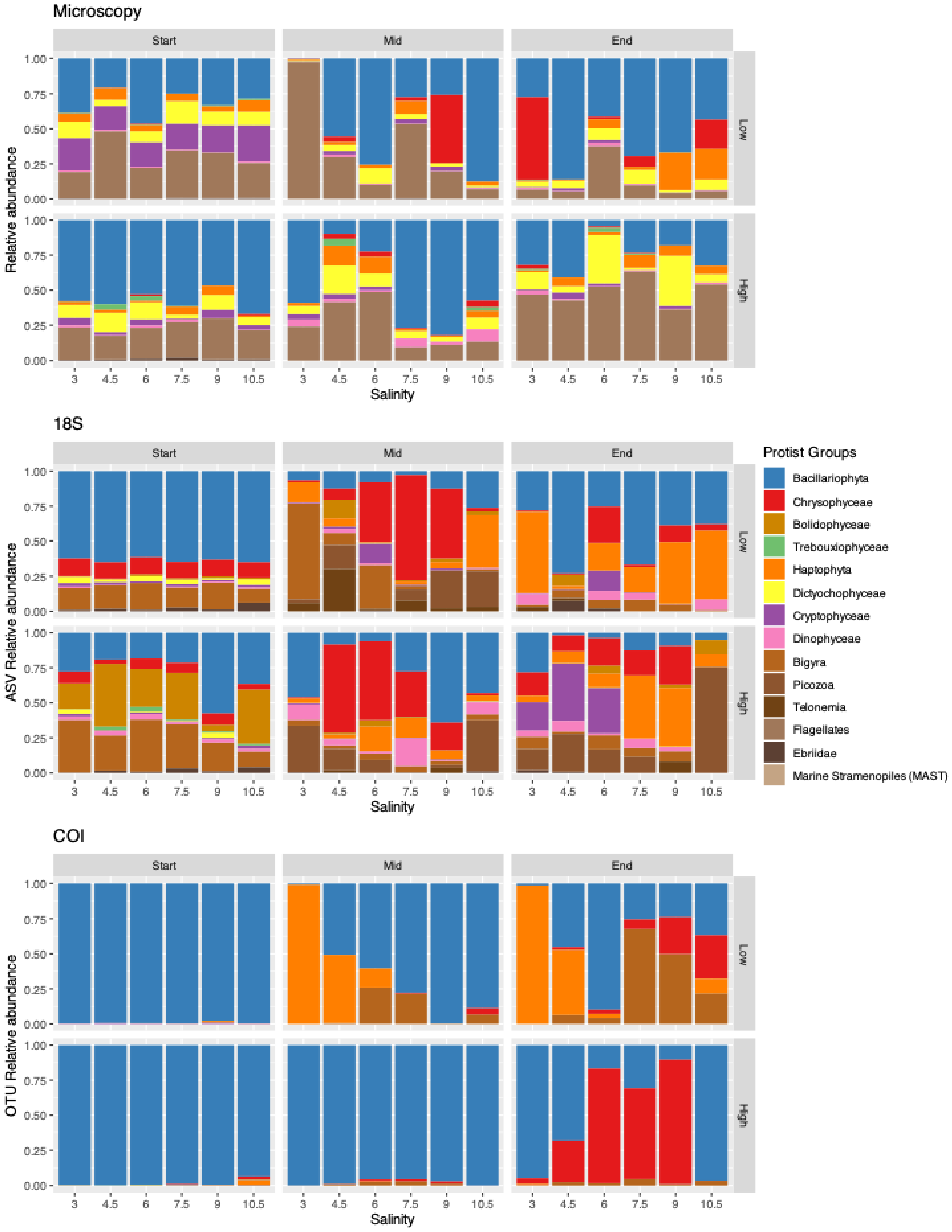

SUPPLEMENTARY FIGURE 1. Protist relative abundance based on microscopy, 18S and COI datasets across salinity levels, temperature treatments (low, high) and time points (Start, Mid, End).

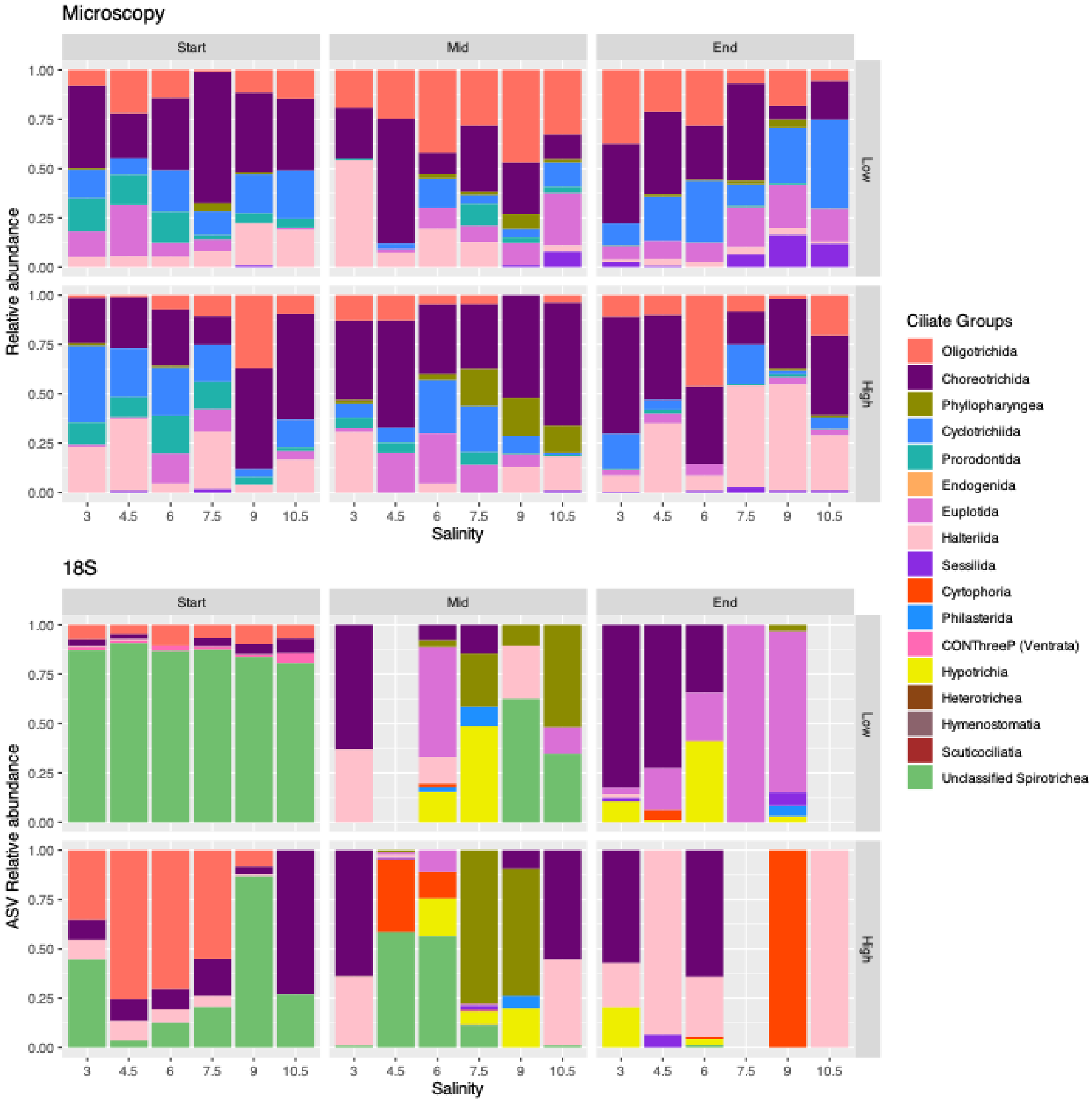

SUPPLEMENTARY FIGURE 2. Ciliate relative abundance based on microscopy, 18S and COI datasets across salinity levels, temperature treatments (low, high) and time points (Start, Mid, End).

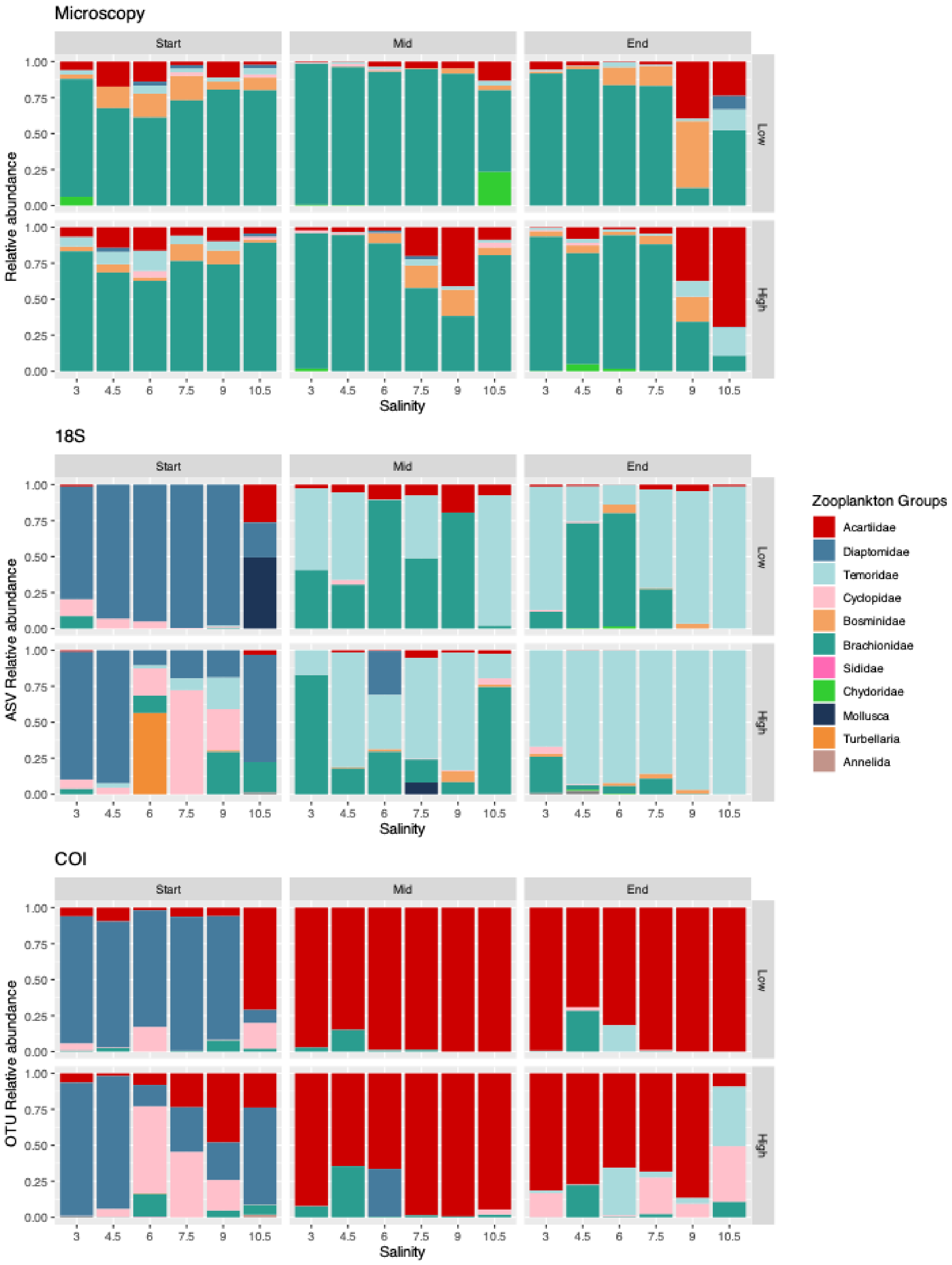

SUPPLEMENTARY FIGURE 3. Mesozooplankton relative abundance based on microscopy, 18S and COI datasets across salinity levels, temperature treatments (low, high) and time points (Start, Mid, End).

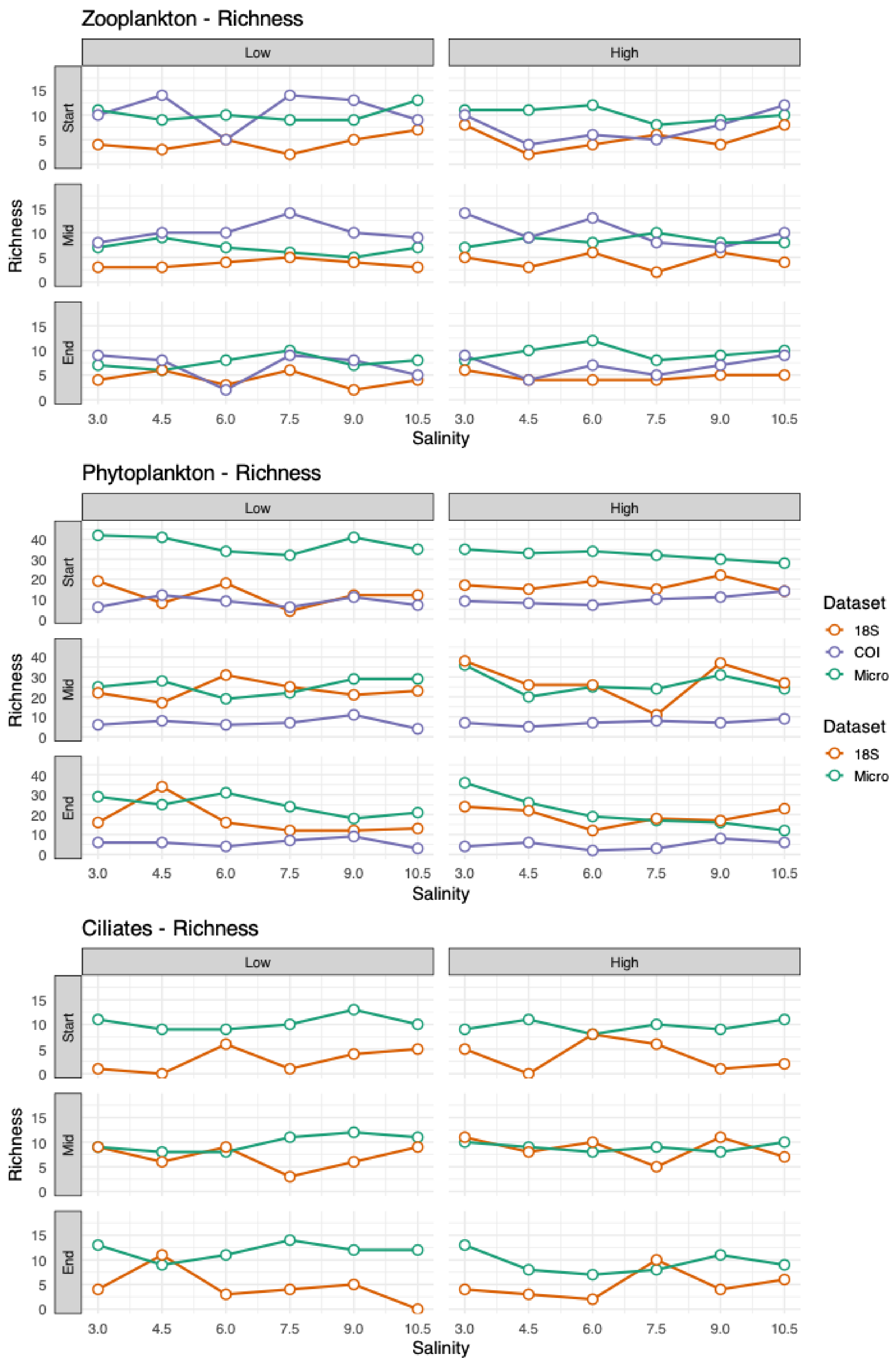

SUPPLEMENTARY FIGURE 4. Richness of zooplankton, phytoplankton and ciliates across salinity levels, temperature treatments (low, high) and time points (Start, Mid, End) for microscopy, 18S and COI datasets.

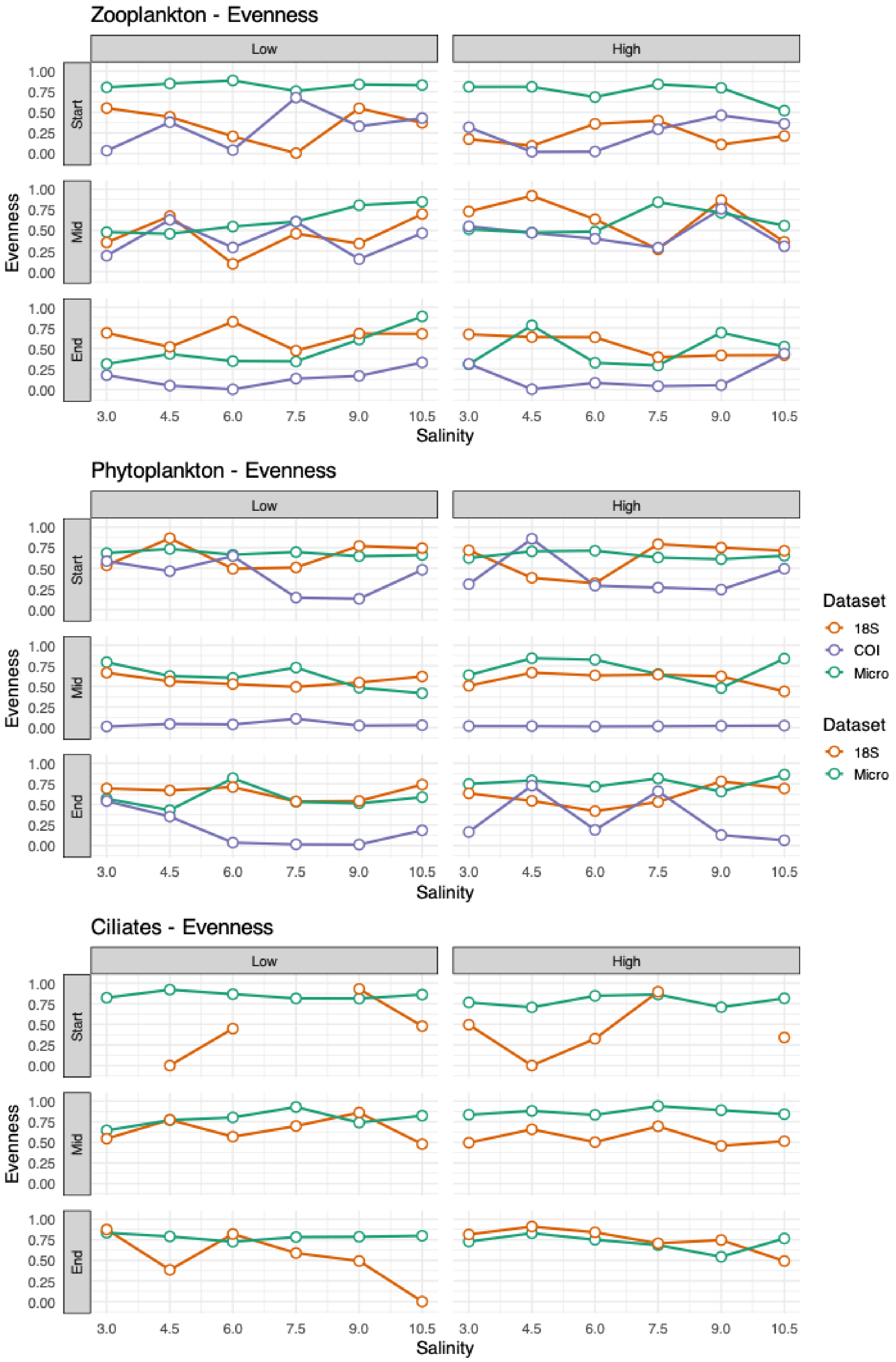

SUPPLEMENTARY FIGURE 5. Evenness of zooplankton, phytoplankton and ciliates across salinity levels, temperature treatments (low, high) and time points (Start, Mid, End) for microscopy, 18S and COI datasets.

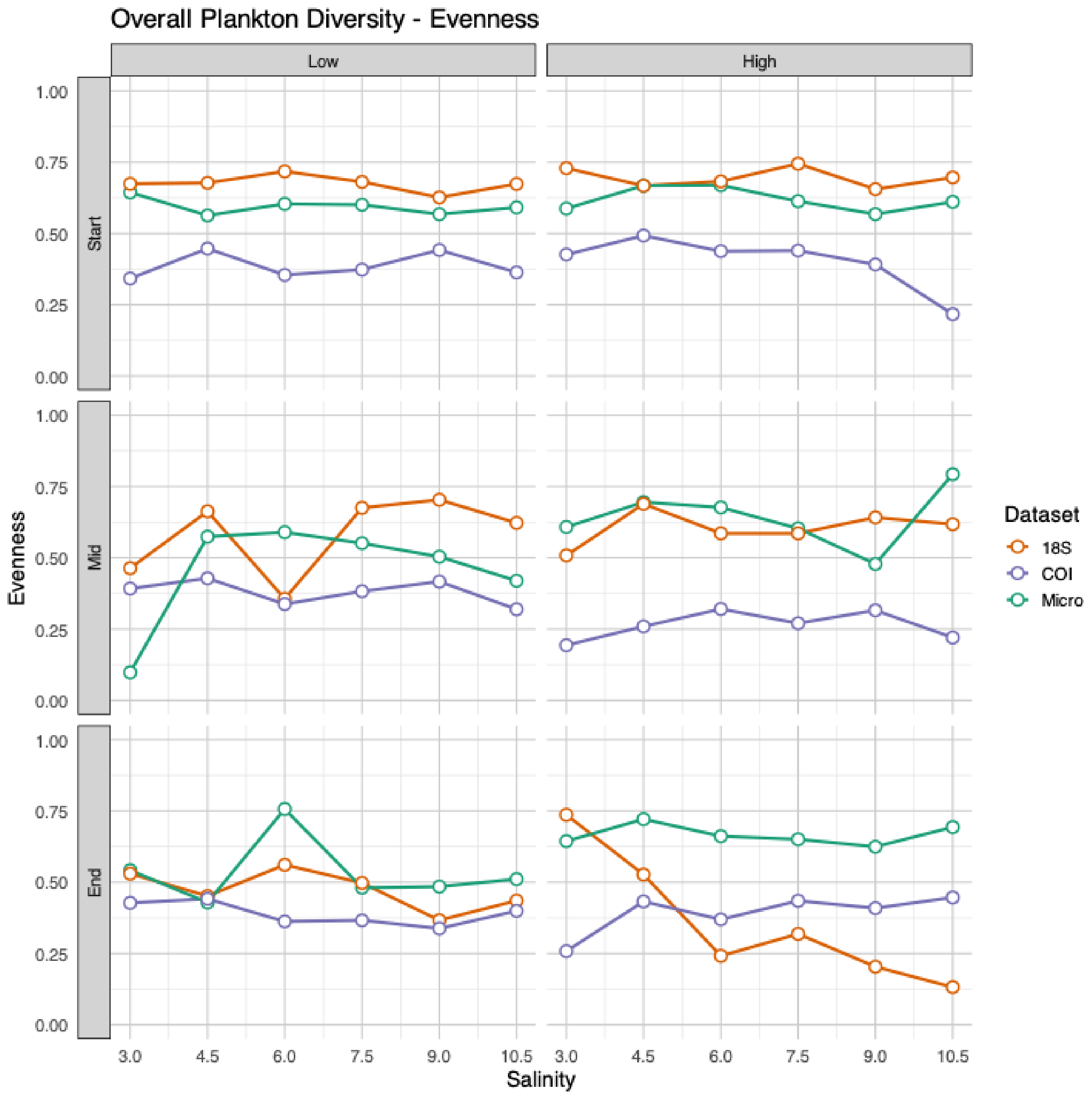

SUPPLEMENTARY FIGURE 6. Overall plankton evenness across salinity levels, temperature treatments (low, high) and time points (Start, Mid, End) for microscopy, 18S and COI datasets.

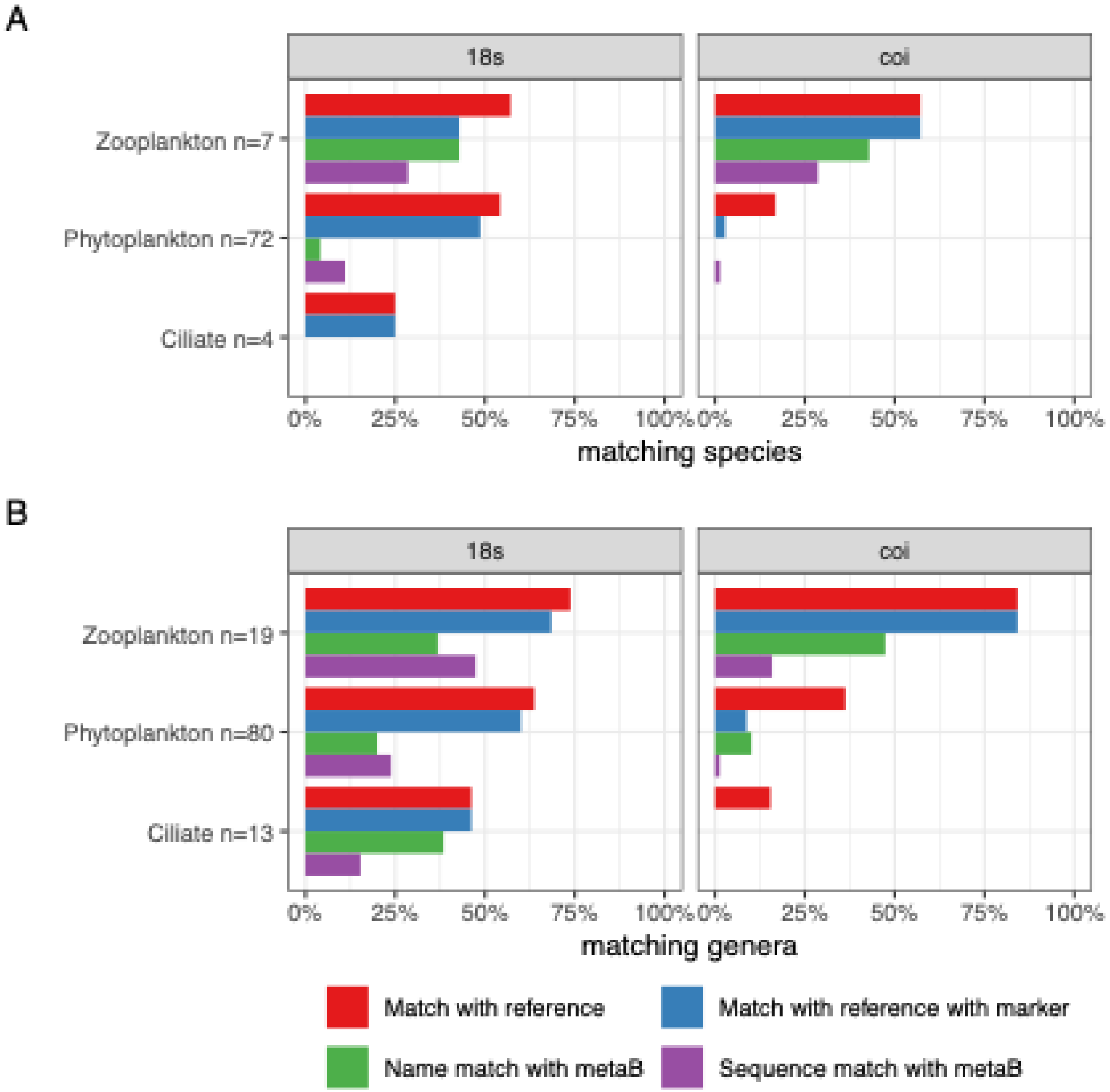

SUPPLEMENTARY FIGURE 7. Proportion of species (A) and genera (B) detected by microscopy that were matched in reference databases (PR2 for 18S, MIDORI2 for COI; red), in marker-specific subsets of the databases (blue), in ASV taxonomic assignment results (green), and in ASV assignments with 100% sequence matches (purple).

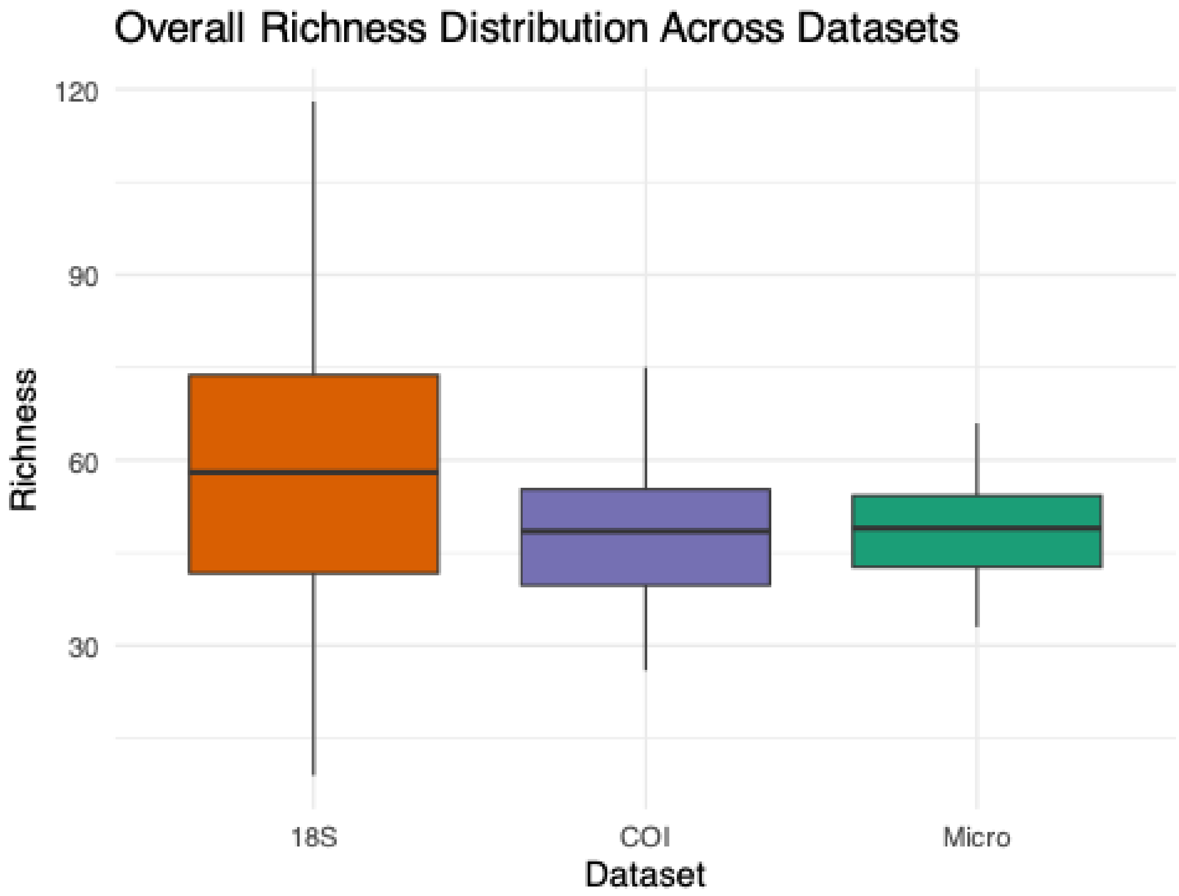

SUPPLEMENTARY FIGURE 8. Distribution of species richness per sample across microscopy, 18S and COI datasets.
